# Divergent Evolution of Human p53 Binding Sites: Cell Cycle Versus Apoptosis 

**DOI:** 10.1371/journal.pgen.0030127

**Published:** 2007-07-27

**Authors:** Monica M Horvath, Xuting Wang, Michael A Resnick, Douglas A Bell

**Affiliations:** Laboratory of Molecular Genetics, National Institute of Environmental Health Sciences, Research Triangle Park, North Carolina, United States of America; National Institute of Genetics, Japan

## Abstract

The p53 tumor suppressor is a sequence-specific pleiotropic transcription factor that coordinates cellular responses to DNA damage and stress, initiating cell-cycle arrest or triggering apoptosis. Although the human p53 binding site sequence (or response element [RE]) is well characterized, some genes have consensus-poor REs that are nevertheless both necessary and sufficient for transactivation by p53. Identification of new functional gene regulatory elements under these conditions is problematic, and evolutionary conservation is often employed. We evaluated the comparative genomics approach for assessing evolutionary conservation of putative binding sites by examining conservation of 83 experimentally validated human p53 REs against mouse, rat, rabbit, and dog genomes and detected pronounced conservation differences among p53 REs and p53-regulated pathways. Bona fide NRF2 (nuclear factor [erythroid-derived 2]-like 2 nuclear factor) and NFκB (nuclear factor of kappa light chain gene enhancer in B cells) binding sites, which direct oxidative stress and innate immunity responses, were used as controls, and both exhibited high interspecific conservation. Surprisingly, the average p53 RE was not significantly more conserved than background genomic sequence, and p53 REs in apoptosis genes as a group showed very little conservation. The common bioinformatics practice of filtering RE predictions by 80% rodent sequence identity would not only give a false positive rate of ∼19%, but miss up to 57% of true p53 REs. Examination of interspecific DNA base substitutions as a function of position in the p53 consensus sequence reveals an unexpected excess of diversity in apoptosis-regulating REs versus cell-cycle controlling REs (rodent comparisons: *p* < 1.0 e−12). While some p53 REs show relatively high levels of conservation, REs in many genes such as *BAX, FAS, PCNA, CASP6, SIVA1,* and *P53AIP1* show little if any homology to rodent sequences. This difference suggests that among mammalian species, evolutionary conservation differs among p53 REs, with some having ancient ancestry and others of more recent origin. Overall our results reveal divergent evolutionary pressure among the binding targets of p53 and emphasize that comparative genomics methods must be used judiciously and tailored to the evolutionary history of the targeted functional regulatory regions.

## Author Summary

The p53 tumor suppressor is a transcription factor that coordinates cellular responses to DNA damage and stress, initiating cell-cycle arrest or triggering apoptosis. Evolutionary conservation is often employed to separate the functional “wheat” from the nonfunctional “chaff” when identifying binding sites of transcription factors like p53. We evaluated evolutionary conservation of 83 experimentally validated human p53 binding sites against mouse, rat, rabbit, and dog genomes, and similarly examined binding sites for two other transcription factors as controls, NRF2 (nuclear factor [erythroid-derived 2]-like 2 nuclear factor) and NFκB (nuclear factor of kappa light chain gene enhancer in B cells), which direct oxidative stress and innate immunity responses, respectively. NRF2 and NFκB binding sites both exhibited high interspecific conservation, indicative of purifying natural selection, but surprisingly, human p53 response elements on average displayed a lack of conservation. Thus conservation is not useful in the prediction of p53 binding sites. After grouping p53 REs by gene ontology, we observed that binding sites in cell-cycle genes like *CDKN1A* displayed high conservation, while p53 binding sites in apoptosis and DNA repair genes showed an unexpected excess of diversity and very little homology with rodent sequences. Overall these results reveal divergent evolutionary pressure among the binding targets of p53 and suggest caution in generalizing about the similarity of regulation of the p53 pathway between humans and rodents.

## Introduction

Since the completion of the human genome, cataloging transcription factor binding sites (TFBSs) has been critical for understanding gene regulation. The use of comparative genomics (evolutionary conservation across species) is often championed as a method to separate the functional regulatory sequence “wheat” from the nonfunctional “chaff” [1]. As the number of mammalian full genome drafts increases, the integration of TFBS predictions with lists of conserved noncoding regions (CNCs) has emerged as a key step in the TFBS identification process [2–4]. If TFBS predictions are contiguous to DNA features that coincidentally have a critical structural role such as maintenance of chromatin organization, the appearance of conservation may be intensified even further.

Although these methods have greatly enhanced our knowledge of the human genome's regulatory repertoire, overreliance on conservation information can potentially exclude genuine binding sites. Since TFBSs are typically small, they can arise by chance in a gene's promoter and therefore may decrease selective pressures to maintain already existing sites [5]. Another wrinkle is that the evolutionary forces that created the conservation blocks may no longer be functionally relevant to humans [6]. Additionally, recent scans for natural selection in human gene coding regions have revealed that distinct biological pathways often are subject to widely different evolutionary pressures [7,8], particularly since mutation rates have been shown to vary across the genome [9]. Genes involved in oncogenesis and tumor suppression have experienced recent selection for mutation in primate lineages [7,8,10]. DNA binding sites of transcription factors are also functional components of these pathways and are likely under similar evolutionary pressures. Indeed, we have focused recently on identifying human single nucleotide polymorphisms that alter the function of transcription factors [11,12]. As a result, we have investigated the assumptions for using mammalian conservation as an obligatory screening step for seeking TFBSs.

The p53 tumor suppressor gene, encoded by the *TP53* master regulatory gene, is a transcription factor that coordinates a network of cellular responses to environmental insults. Over half of human cancers have a mutation in the p53 protein or one of its partners [13]. The p53 protein is estimated to have several hundred transregulation target genes that affect pathways including apoptosis, DNA damage repair, and cell-growth arrest [14]. As a result, p53 target genes are highly sought-after drug targets for halting cancer progression. According to in vitro experiments, the p53 protein binds specifically to a palindromic consensus sequence, RRRCWWGYYY(N_0−13_)RRRCWWGYYY [15], with nearly all REs containing at least one mismatch; in vivo results have suggested that the spacer region may be much smaller [14,15]. The sequence is typically located within 5,000 bases of the target gene's transcriptional start site, and p53 either induces or represses expression upon p53 binding [16,17]. One feature of p53 that confounds the discovery of novel transregulated genes is that while some binding sites match the expected consensus sequence quite well, others can be consensus poor and yet are both necessary, and sufficient, to transactivate a gene [18]. A recent study has suggested that the “rules of engagement” for p53 REs may differ based on the activated pathway, particularly in the apoptosis and cell-cycle–related systems [19]. Thus, we have used cross-species conservation to examine if these groups of elements exhibit distinct conservation profiles.

To evaluate the utility of comparative genomics approaches in the identification of potential p53 target REs, we gleaned the literature for a high quality set of bona fide p53 REs to estimate the degree of conservation between humans and other mammals. To relate the *TP53* system to other master regulators, we compare its binding site conservation to those of the transcription factors encoded by two other genes: *NFκB* (nuclear factor of kappa light chain gene enhancer in B-cells)*,* central to inflammation responses, and *NFE2L2,* which encodes NRF2 (nuclear factor [erythroid-derived 2]-like 2 nuclear factor), a regulator of oxidative stress. Their repertoire of interactions is expected to be highly preserved throughout the mammalian lineage. The NFκB transcription factor is a heavily studied biological switch of the inflammation, apoptosis, and immune responses [20,21]. It binds the consensus sequence GGGRNNYYCC [22,23], and its signaling system is highly conserved even when examined in invertebrates [21,24]. NRF2 binds antioxidant REs (consensus sequence = TMANNRTGAYNNNGCRWWWW [25]) that are comparable in size to those of p53, show high levels of conservation [26], and are found in the promoters of genes that confer protection from oxidative stress and chemical carcinogens [27]. Mouse models of Nrf2-dependent response to oxidative and electrophilic insults have been used to study function [28,29]. Additionally, the Nrf2 pathway in zebrafish operates similarly to humans and underscores the likelihood of high conservation in regulatory binding sites [30]. Because the NFκB and NRF2 binding sites were determined to be highly conserved, these two sets of TFBSs serve as positive controls in estimates of conservation. Our comparative genome analysis, which includes a coincident evaluation of sampled promoter sequences and coding region sequence, reveals that mammalian conservation does not apply to p53 target REs in general. However, among subgroups of target genes we observe purifying selection acting on a number of p53 binding sites, including many cell-cycle–related genes, while rodent to human homology is lacking for p53 REs in apoptosis-related genes.

## Results

### Conservation of TFBSs

**Figure 1:**
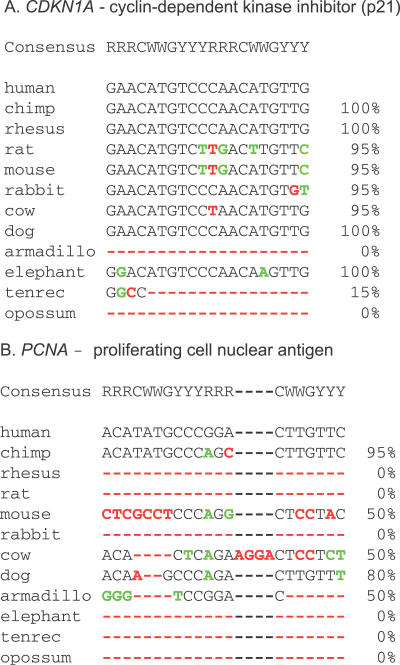
Examples of Calculated Interspecies Conservation Scores for TFBSs Multispecies alignments for p53 binding sites in the (A) *CDKN1A* and (B) *PCNA* genes are shown. Mismatches (green) from human that do not alter the p53 consensus motif, RRRCWWGYYY(N_0−13_)RRRCWWGYYY, do not penalize the percent sequence identity, whereas consensus-altering mismatches and insertion/deletion events do (red). For the p53 binding sites, spacer elements are in gray and are not considered when calculating percent identity. R = G or A; W = A or T; Y = C or T.

The literature was scanned for validated p53 (83, Table 1), NFκB (21, Table 2), and NRF2 (21, Table 3) binding sites associated with human genes. Human genome coordinates were located and then referenced within global multiple alignments held at the University of California Santa Cruz [UCSC] (California, United States) genome browser website [31] to find their corresponding locations in eleven other mammals. Using these global alignments, percent sequence identity was calculated for each of the 125 binding sites across the eleven mammals, with the calculation adapted to reflect consensus sequence degeneracy, since every model RE had positions where one or more of the four nucleotides could be tolerated. Also, the p53 RE was unique in that the spacer region between the two half sites could be any size or sequence up to thirteen bases. We therefore calculated sequence identity by omitting the p53 spacer region and ignoring mismatches in the alignments that still fit the TFBS consensus (*CDKN1A* and *PCNA* examples shown in Figure 1, others in Figure S3).

**Table 1:**
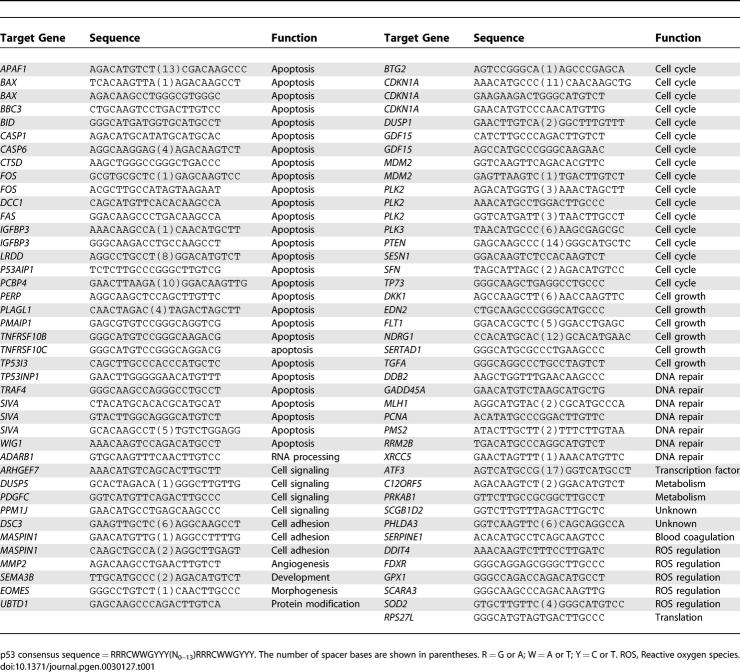
Validated Human p53 TFBSs

**Table 2:**
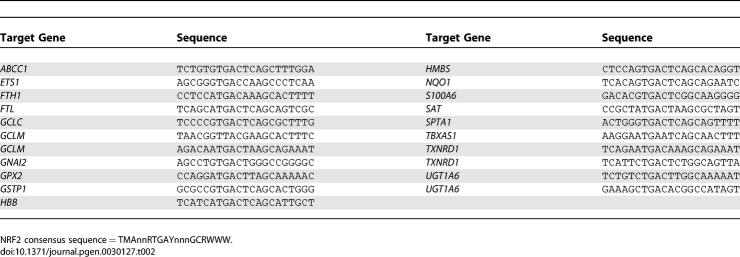
Validated Human NRF2 TFBSs

**Table 3:**
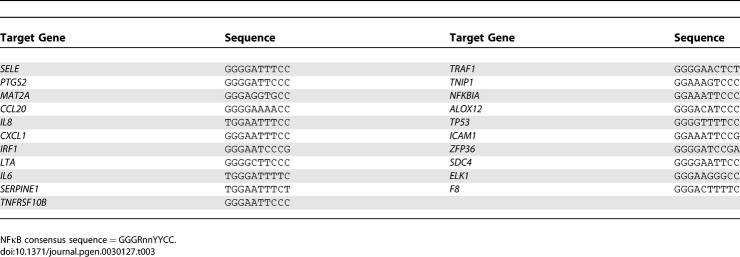
Validated Human NFκB TFBSs

**Figure 2:**
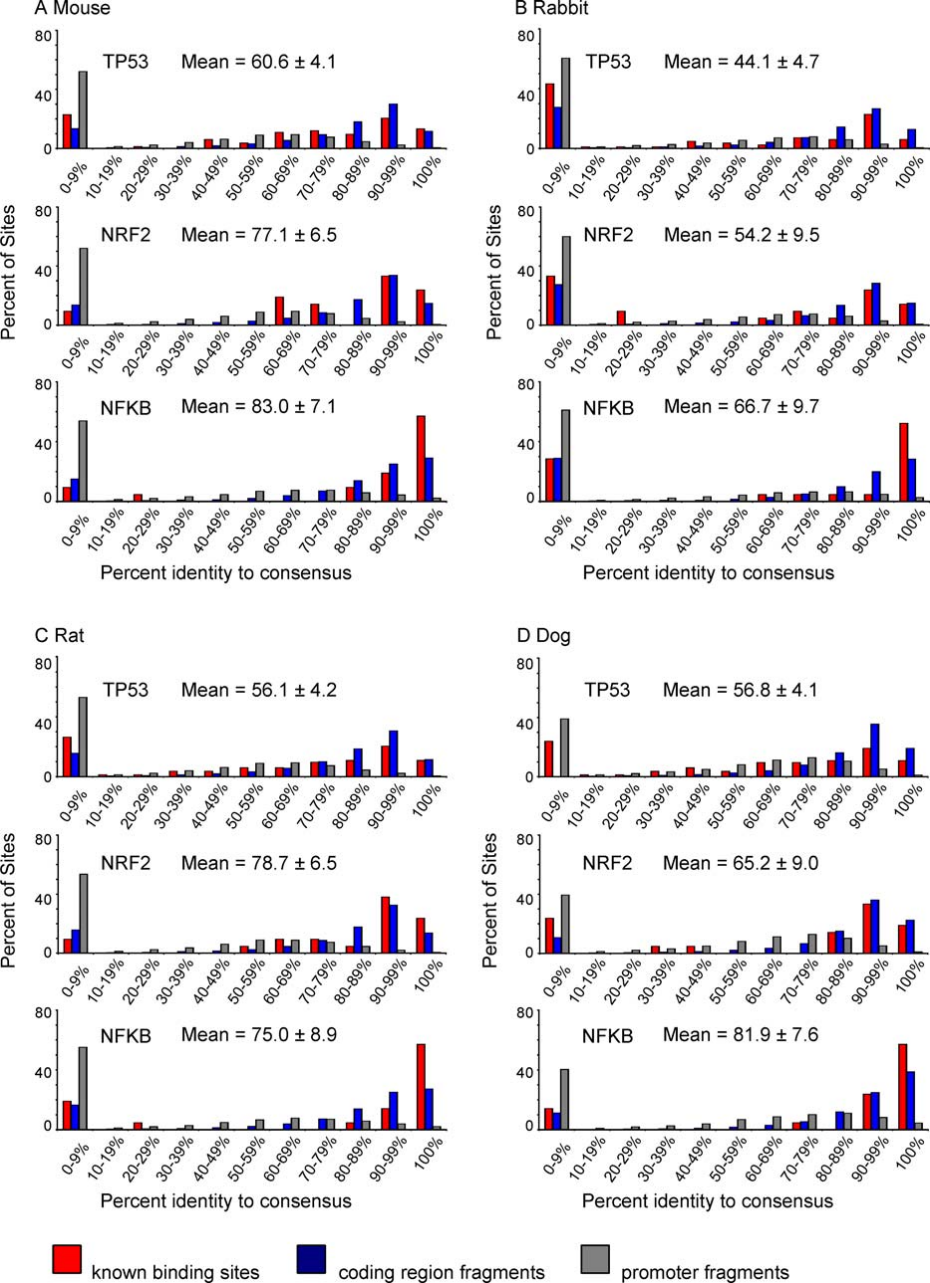
Distribution of Human-to-Mammal TFBS Conservation Scores Graphed for each (human-to-mouse, -rabbit, -rat, and -dog) comparison in (A–D) are: the percentage of all TFBSs from each gene group (p53, 83 REs; NRF2, 21 REs; or NFκB, 21 REs) that fall within the x-axis conservation bins (red bars); data for randomly sampled fragments from human coding regions (blue bars); and gene promoters (gray bars). Coding regions (representing sequence under purifying selection) and randomly sampled promoter regions (representing sequence under more neutral selection) were sampled 1,000 trials; error bars are narrower than the line art. Averages and standard errors for the sets of known TFBSs are reported above each graph.

Figure 2 plots the conservation distribution for each set of human TFBSs across mouse, rabbit, rat, and dog. Although comparative data relating the chimpanzee and rhesus monkey were also analyzed, we observed, as expected, that these species were too evolutionary close to humans to be informative (Figure S1). For example, nearly any human sequence was in excess of 95% conserved in these two primates. Also graphed in Figure 2 are the results for sets of DNA sequence fragments randomly chosen from promoter (gray) and protein-coding (blue) regions. This allows TFBS conservation levels to be viewed in context of the evolutionary pressures exerted on other genomic sequences. The poorly conserved element shown in Figure 1 *(PCNA)* would fall in a lower percentage bin (i.e., be on the left side of the graph), as is the case for the randomly chosen promoter sequences. The promoter fragments are representative of the background genome sequence in which most TFBSs reside. On the other hand, if TFBSs were very well conserved, then the distribution would be right-shifted, as is the case for the protein-coding region fragments (blue). To use conservation as a metric to separate true binding sites from the rest of the genome, their conservation should be significantly greater than that of randomly chosen promoter regions. The spike of TFBSs at 0%–9% identity in each panel represents species-specific sites that are essentially not present in the other mammals.

For each of the human-to-mammal comparisons in Figure 2, *NFκB* and NRF2 sites produced identity distributions that appear very similar to distributions from the coding region fragment group (many sites with 90%–100% identity), which was representative of genome sequence under high purifying selection. This strongly suggests that *NFκB* and NRF2 sites may be under purifying selection. The human-to-mammal p53 site comparisons, on the other hand, produced conservation profiles in each species that have a high frequency of sites at zero percent identity and fewer with 90%–100% identity. This distribution is similar to the distribution obtained from randomly sampled promoter fragments (gray), which we used to represent genome sequence under neutral selection. In mouse the p53 RE identity distribution was correlated with the promoter fragment identity distribution while NFκB and NRF2 showed less correlation with the promoter distribution (Table S3).

Since p53 sites as a group were observed to have as many interspecific substitutions as the background genomic sequence, use of conservation level to predicting bona fide sites would not be effective. However, this result could be due to the fact that the set of 83 p53 TFBSs actually represents two or more subsets of p53 REs with distinct conservation profiles. A recent study hypothesized that the sequence requirements of p53 REs may differ based on the activated pathway such as apoptosis, DNA repair, cell-cycle checkpoints, or cell-growth arrest [19]. We therefore investigated if low and high percent identity values would apportion with p53 REs grouped by function, thereby detecting evolutionary divergence between p53 pathways.

Among these 83 p53 REs, we carried out analysis of the two largest subgroups (Table 1), apoptosis-related (*n* = 29) and cell-cycle/cell-growth–related (*n* = 23), on the basis of observations of Qian et. al [19]. Average percent identity to a consensus sequence was calculated for each of the transcription factor groups, including the p53 subsets, and compared via a two-tailed t-test assuming unequal variances between the datasets. The results are displayed as odds ratios (OR) in Table 4, and OR values represent the odds that one type of human TFBS (columns) will be found as more conserved than a second TFBS type (rows) in comparison with other mammalian species. Ratios less than 1 (e.g., p53 apoptosis compared to all p53 sites) suggest lower conservation of the TFBS in the row. Among all species, the relative conservation levels of NFκB compared to NRF2 sites were similar and the *p*-values for difference were not significant. NFκB sites were significantly more conserved than the entire set of p53 sites in mouse and dog, while NRF2 sites were significantly more conserved than all p53 sites in mouse and rat. The magnitude and statistical significance of the differences between sequence motifs in Table 4 was greatest when comparing either NRF2 or NFκB and the apoptotic p53 sites. For example, in each species, the ORs for relative conservation between either NRF2 or NFκB and apoptosis genes were all high (OR > 3.0) and at a significance of at least *p* < 0.019. On the other hand, the mean conservation level of the cell-cycle–regulating p53 REs were not statistically different from the NRF2 or NFκB sites. These observations imply that the mean p53 conservation level for all elements is really a combination of the effect of the two ontological subgroups.

**Table 4:**
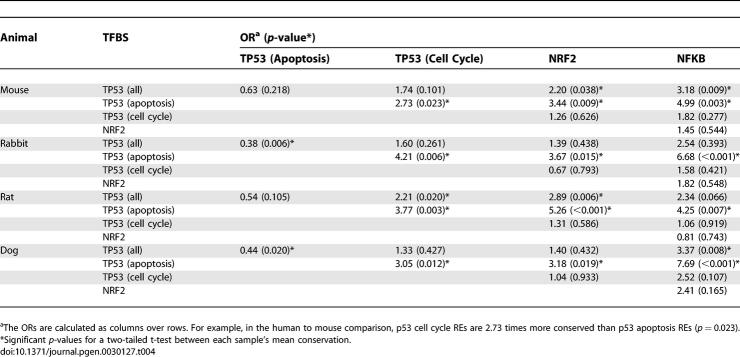
Comparison of Mean TFBS Conservation Values (Mouse to Human, Rabbit to Human, Rat to Human, and Dog to Human)

It has been proposed that an alternative method for evaluating a DNA fragment's conservation level is to ask whether it sits within a block of conservation [6]. All bona fide TFBSs examined in this study were matched against the “most conserved” track of the UCSC genome browser to ask whether a significant proportion fell within conserved blocks. Only 19.2% of all p53 REs mapped to these regions, while 52.8% of NRF2 and 57.1% of NFκB TFBSs could be colocated (Table 5). We also assessed the conservation of the randomly chosen promoter sequences according to this block method and used a two-tailed binomial test to calculate statistical significance. Intriguingly, all TFBS groups mapped to more blocks than the random promoter sequences except for the apoptosis subgroup of p53 REs. These data mirrored the percent identity conservation metric and again underscored that the apoptotic-regulating p53 TFBSs may not have been under purifying selection throughout mammalian evolution.

**Table 5:**
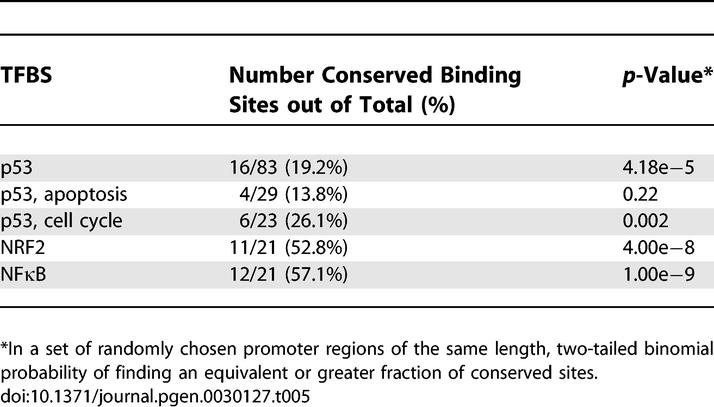
Mapping of Human TFBSs to Hidden Markov Model-Generated Conservation Blocks

Inspection of the individual alignments between human p53 REs and mouse reveal that 38% (11/29) apoptosis elements and 9% (2/21) of cell-cycle element could not be aligned with the multiz global alignment tool and thus showed zero identity. Thus for human p53 REs in genes such as *BAX, FAS, PCNA, CASP6, SIVA1,* and *P53AIP1* we observed little, if any, similarity with rodent sequences, and these nonaligned sequences (zero identity) strongly impact the calculations we have made.

### Predictiveness of Mammalian Conservation for TFBSs

Although it was informative to know how well human p53 TFBSs are conserved relative to other regulatory motifs, an aim of this study was to probe the utility of comparative genomics for authenticating binding sites predicted by computational methods. Receiver operator characteristic (ROC) curves were employed to demonstrate the sensitivity of TFBS prediction when qualified by conservation information. ROC curves are traditionally used to measure the quality of a binary classification algorithm, as a discrimination threshold is varied. Area under a ROC curve provides a visual representation of how well the conservation metric can classify the sets of bona fide TFBSs as true positives. For example, the area can be interpreted as the probability that when both a bona fide TFBS and a random promoter sequence of equal lengths are chosen at random, the decision function (conservation in a species) assigns a higher value to the bona fide TFBS. A perfect decision function would generate a curve with an area of 1, meaning that 100% sensitivity was obtained (i.e., all true positives were found), and 100% specificity was reached (i.e., no false positives were generated). If conservation predicted TFBS authenticity no better than random chance, a line at 45 ° to the x-axis would be generated that bisects the ROC space (area under the curve [AUC] = 0.5), because as the threshold is raised, equal numbers of true and false positives compose the chosen set of TFBSs. A ROC curve that fell below this diagonal would indicate that conservation consistently predicted poorly, meaning that one should employ the lack of conservation as a decision classifier to authenticate TFBSs.

**Figure 3:**
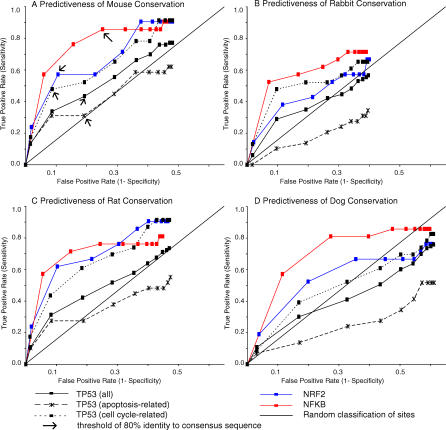
ROC Curves for Four Mammalian Comparisons Indicate the Ability of Different Interspecific Comparisons to Identify Bona Fide Human TFBSs (A–D) Using a dataset of experimentally verified human TFBSs and randomly chosen promoter sequences from human genes, the true positive and false positive prediction rates were calculated given conservation thresholds that classify a TFBS as authentic or spurious. Each data point on a curve represents a different conservation threshold for comparison in the indicated species. In (A) arrows indicate the 80% identity threshold. For NFκB 80% identity with mouse is highly predictive for human. Prediction of apoptosis sites are similar to random classification (diagonal line).

When TFBS conservation was evaluated as a TFBS predictor in each of the four mammals, bona fide NRF2 and NFκB sites were consistently well predicted, whereas the ROC curve describing all p53 sites approached the random diagonal (Figure 3A–3D). The latter implies that conservation analysis in these model organisms cannot enhance p53 binding site discovery, for the predictive capacity is only slightly better than random. For example, if a cutoff of 80% identity to mouse was employed as the rule for choosing p53 binding sites, only 43% of real p53 REs would be found, and 19% of the selections would be false positives (Figure 3A). We were concerned that ascertainment bias (e.g., the presence of spurious REs) in this large set of p53 sites might affect our findings. However, the predictivity level for any of these species does not change appreciably even when the p53 RE list is restricted to only the 30 best-characterized sites (Figure S2). In contrast, for NFκB an 80% mouse conservation threshold would allow discovery of 86% of real NFκB sites with a 25% false positive rate (Figure 3A). As a result, human/mouse multiple sequence alignments are highly useful for identifying novel NFκB sites but not so for p53 motifs. This conclusion is reiterated in all four model organisms by statistical evaluation of the AUC calculations (Table 6). p53 curves had smaller AUCs compared to those for NFκB and NRF2.

**Table 6:**
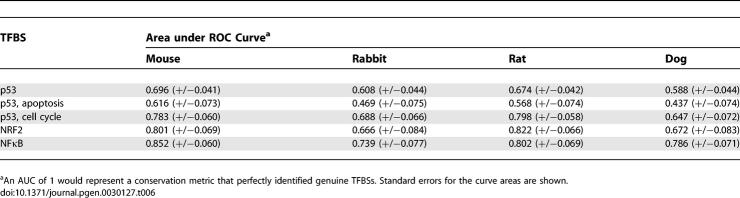
Efficacy of Mammalian Conservation to Predict Human TFBSs

A recent study noted that although the spacer region between half sites for p53 REs can be zero to13 bases, small spacers were overwhelmingly preferred in a distribution of spacer length derived from genome-wide chromatin immunoprecipitation experiments [14]. This suggests that REs with large spacers might not be valid, and we hypothesized that if ROC curve analysis was restricted to only p53 REs with small spacers (presumed higher quality), perhaps much greater conservation would be observed. We examined the set of p53 REs having two or fewer spacer nucleotides between half sites and observed no increase in conservation (Figure S2). Not only was there no improvement in TFBS prediction sensitivity for this subset, but the ROC areas were visibly greater for rat and rabbit comparisons in the inverse set of REs (i.e., 3+ spacer bases). Thus, as judged by conservation, p53 RE spacer region length could not be considered a measure of RE quality.

We then examined p53 RE conservation in light of gene ontology. When p53 REs were subdivided based on functional class, the sensitivity of interspecific conservation to predict cell-cycle/cell-growth sites improved considerably, approaching that for the NFκB and NRF2 targeted genes (Figure 3). The p53 apoptotic REs (Figure 3, dashed line), on the other hand, showed a dramatically different conservation profile. In the case of the mouse (AUC = 0.616) and rat (AUC = 0.568), the ROC curve hovered just above the random line, which indicates a lack of sensitivity. For two species (rabbit, AUC = 0.469 and dog, AUC = 0.437), the sequence identity metric had an apoptosis RE discovery rate worse than random prediction. This suggests that the functional, apoptotic p53 binding sites are less conserved than randomly sampled sequences in gene promoters. This phenomenon was also observed when ROC curve analysis was carried out in other distant mammalian species (tenrec, armadillo, elephant, and opossum) (unpublished data).

One explanation for this provocative result could be that apoptotic p53 sites might actually display a slightly different consensus p53 binding site than that reported in the literature. Perhaps a better-fitting consensus would improve conservation. We aligned all p53 sites (83), apoptotic (29), and cell-cycle (23) p53 sites (Figure S3) and generated sequence logos [32] (Figure S3A–3C) to identify improved patterns, but while there are small differences, none fit better than the existing consensus of RRRCWWGYYYN_0−13_RRRCWWGYY. Likewise, simply permitting any nucleotide at the least compositionally biased positions in this p53 RE subset (bases 2, 8, 10, and 11 of the p53 consensus) did not improve the area under the ROC curve (Figure S2). These data emphasize not only that conservation analysis cannot improve identification of certain TFBSs like p53, but also that subclasses of the same binding site may reflect distinct evolutionary profiles.

### Sequence Diversity as a Function of Position in the Consensus Sequence

**Figure 4:**
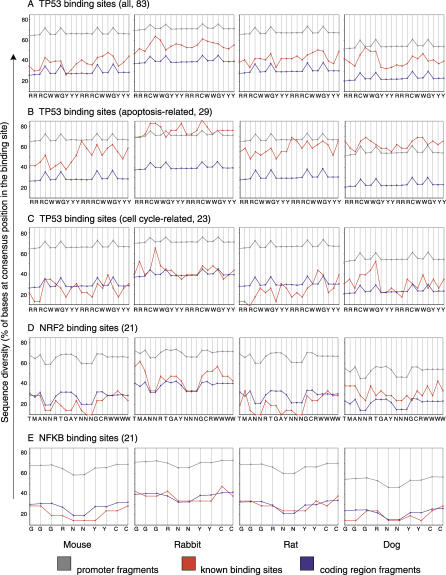
Positional Diversity of Human TFBSs Compared to Four Species (A–E) Sequence diversity is calculated as the percentage of aligned bases at a certain position that vary from the accepted transcription factor consensus sequence, which is listed along the x-axis. The diversity levels of randomly chosen promoter sequences (gray) and randomly chosen coding region (blue) sequences (1,000 trials, error bars are narrower than the data points) are plotted so that level of genuine TFBS diversity (red, known sites) can be viewed in a genomic context. The shape of each plot reflects the level of degeneracy of the consensus at each nucleotide position (e.g., gray line, the p53 consensus has C and G at fixed positions creating peaks in the samples from the promoter)

A second approach was used to detect if conservation differed among nucleotide positions within the binding site. That is, could we observe heightened human-to-mammal interspecific substitutions or “sequence diversity” at particular locations within each TFBS consensus sequence? To accomplish this, we aligned all TFBSs within each group (p53, NRF2, or NFκB) and calculated the positional sequence diversity, which was the percentage of aligned bases at each position that varied from the accepted consensus sequence (Figure 4). For example, in Figure 4A, the first position in the p53 consensus sequence differed from a purine base (R) at the equivalent position in the mouse in 35% of all p53 TFBSs, while positional diversity for randomly sampled promoter sequence was 63% and that for coding region sequence was 25%. Highly conserved sequence would be plotted lower on the y-axis (less diversity) as displayed by the coding region line (blue), while less conserved sequence would exhibit high diversity and appear near the top of the graph (e.g., gray, random promoter sequences). Small peaks observed in the promoter and coding region plots reflect degeneracy of the consensus sequence, with more degenerate positions exhibiting less calculated diversity. Patterns of the promoter and coding sequence lines are highly similar across species in Figure 4 except for being shifted on the y-axis. This was an expected feature of the data since these control curves were plotted as the average result of 1,000 trials of sequence fragment sampling across the human genome. When examining the population statistic of a large number of fragments, the average coding or promoter region fragments will exhibit similar transversion and transition mutation rates across species, which are visualized in these patterns.

Figure 4D and 4E demonstrate that the positional sequence diversity of NRF2 and NFκB sites mirrored coding region sequence diversity across all species, as expected from the previous conservation analysis (Figure 3). Figure 4C shows a similar effect, with p53 cell-cycle–related sites displaying low sequence diversity. Intriguingly, the apoptotic-related p53 binding sites (Figure 4B) showed levels of sequence diversity that often met or exceeded those of the background promoter sequences. In rabbit and dog these apoptotic p53 binding sites have diverged so much that they may have lost function or could be under positive selection for mutation. The differences in positional sequence diversity between the two p53 RE subgroups were all highly significant (two-tailed paired t-test assuming unequal variances: dog = 1.4e−13, mouse = 4.3e−12, rabbit = 2.0e−17, and rat = 2.0e−15). These dramatic results indicate again that these different classes of p53 binding sites may be subject to widely dissimilar sequence constraints.

## Discussion

The wide variety of genes transcriptionally regulated by p53 highlights the pleiotropic role of this master regulatory protein in many different biological pathways. Here, we addressed the conservation of human p53 binding sites across several mammals commonly used as experimental models. Examining global alignments of established human p53 binding sites, we found that common comparative genomics methods do not generally enhance p53 binding site prediction, although they can for NFκB, NRF2, and a subset of p53 target genes involved in cell-cycle regulation. This apparent lack of conservation for many functional human p53 binding sites suggests that regulation of the p53 response network may be fine tuned for the needs of each species. By comparing sequence conservation between two p53 pathways, we have detected differences in the evolution of their regulatory elements. In particular, numerous functional human p53 REs in apoptosis-regulating sites, as well as the surrounding local sequence, show little homology to rodent sequences, suggesting that this ontology may have been shaped by primate-specific selection pressures that have resulted in turnover (loss or gain) of binding sites. This is supported to some degree by the very high mean sequence identity for all p53 REs between human and chimpanzee or monkey (Figure S1). However, turnover cannot be easily addressed by the global alignment method. For example, a short species-specific rearrangement such as an insertion of a repetitive DNA element (e.g., SINE, LINE, etc.) that contains a RE would not globally align and would show zero identity across species but might maintain functional response across species.

There were also seven DNA repair-regulating p53 REs in our dataset. Although there were not enough p53 RE sites to perform a statistically significant analysis, the average percent identity to the consensus sequence was similar to that of the apoptosis-related subset for human-to-rodent comparisons. These findings are significant considering the efforts to functionally model human p53 responses in the mouse (including cell cycle, apoptosis, and DNA repair) [33–35].

Complex molecular events (reviewed in [35]) regulate both p53 levels and activity prior to the transregulation of cell-cycle arrest and apoptosis genes. This results in large increases in p53 availability for binding to REs. Presumably the strength of p53 binding to a given target sequence has the effect of tuning regulation of the components of the p53 network within a species. Our data suggest that regulation of some p53 pathways, including apoptotic and DNA repair genes, may differ between humans and other mammalian species. Not only are REs in apoptotic and other genes different from cell-cycle genes in rodents, but they appear to differ from rabbits and dogs as well (Figure 4B). This unexpected excess of sequence diversity for apoptotic elements could be explained by recent positive selection in all of these species. Support for this comes from an evolutionary analysis of a functional, yet poor, consensus-matching p53 RE in the apoptotic gene *PIG3*. This study revealed that *PIG3* became p53 responsive only recently, during primate evolution [10] and is consequently only present and functional in apes and humans.

A recent emergence of primate-specific apoptosis p53 RE sites could explain the large number of interspecific differences identified following alignment to their orthologous mouse, rat, rabbit, or dog sites (Figure 4). Dermitzakis and Clark [36] observed a similar phenomena while surveying a broad number of TFBSs and concluded that a large percentage of apparently functional human sites were not functional in rodents (and vice versa). The authors suggest that loss and gain of TFBSs has been commonplace in both rodents and humans. On the other hand, the p53 protein itself has changed very little between species. Mouse and human p53 proteins are 85% identical and show equivalent transactivation of human apoptotic and cell-cycle REs in a yeast-based system [37]. The DNA binding domain of the p53 protein has near 100% homology across all mammalian species indicating strong purifying selection to maintain DNA binding function. Our data indicate that cell-cycle REs are also being maintained by purifying selection, while the evidence suggests that divergent positive selection has occurred among REs of apoptosis genes. The evolution of apoptosis-related p53 binding sites has strong biological plausibility, as it seems likely that such modifications could profoundly affect how a species responds to environmental stress and cellular damage. With exposure to DNA-damaging agents being a common environmental feature throughout mammalian history, selection pressure and the evolution of systems to maintain genome stability could be quite different in rodents and primates. For example, it was recently shown that the *Spalax* (mole rat), which lives its entire life underground, has a p53 protein with a very limited ability to induce several well-known human apoptotic genes in reporter assays. It is, however, quite capable of transactivating cell-cycle arrest genes [38]. The adaptation of the *Spalax* p53 response to a dramatically different environment underscores how separate pathways jumpstarted by the same transcription factor can have distinct evolutionary signatures.

Cross-species analysis of p53-regulated genes in relation to biological function is largely absent. Thus it is unknown whether any preservation of functionality in apoptosis-related p53 binding sites exists, or if divergence and positive selection have created uniquely primate response characteristics. We are currently evaluating how p53 RE variation across species affects binding and transactivation in a functional model system and have observed that some weak binding REs show high conservation (D. A. Bell, unpublished data). Other aspects of p53 pathways may evolve, such as the proteins that regulate the availability or quantity of active p53 protein, so that the sequence and binding affinity of affiliated binding sites could be coevolving with such changes.

This study makes several suggestions for computational analysis of p53 REs and regulatory sequence in general. Since these binding sites seem to be experiencing much short-term evolution and turnover, comparative genome analysis in a panel of old and new world monkeys, “phylogenetic shadowing,” may be a promising direction to enhance prediction accuracy [1]. Secondly, among those p53 REs exhibiting high conservation, mutations or polymorphisms that alter such sequences may be significant [35]. A key practical point is that if comparative genomics methods are used to identify putative functional regulatory regions, one should ensure that the choice of comparative species data is relevant to the selection pressure on the motifs of interest. For the p53 pathway, predictions based on mouse- or rat-to-human will not only generate a large excess of false positives, but many bona fide REs will be missed.

Overall, conservation analysis may be a convenient measuring stick for regulatory element function, but we have shown that it must be used with caution and may depend on the TFBS category being analyzed. A reduction in evolutionary conservation in p53 regulatory elements is likely due to species-specific selective pressures acting on the distinct biological differences among p53-regulatory pathways.

## Materials and Methods

TFBSs with experimental support were located in the literature (see Table S1). Genome coordinates (National Center for Biotechnology [NCBI] 35.1, May 2004 release) were located using BLAT [39] searches against the human genome within the UCSC genome browser (Table S2) [31,40]. If a TFBS could not be found in the genome, it was removed from analysis, which left 83, 21, and 21 binding sites for p53, NRF2, and NFκB, respectively. The UCSC “multiz17way” conservation track [31,40,41] provided a 17-way multiple sequence alignment between current releases of the Homo sapiens genome and eleven additional mammals: Pan troglodytes (chimpanzee, November 2003), Canis familiaris (dog, May 2005), Mus musculus (mouse, May 2004), Rattus norvegicus (rat, June 2003), Macaca mulatta (rhesus monkey, January 2006), Monodelphis domestica (opossum, June 2005), Bos taurus (cow, March 2005), Echinops telfairi (tenrec, July 2005), Loxodonta africana (elephant, May 2005), Oryctolagus cuniculus (rabbit, May 2005), and Dasypus novemcinctus (armadillo, May 2005). This alignment set was used to find the corresponding locations of each TFBS within each genome. The accuracy of these alignment regions were manually inspected and verified by both confirming similar local gene organization as well as referencing independently generated paired human–mammal alignments (UCSC tables netMm7, netMonDom1, netBosTau2, netRn4, netCanFam2, netRheMac2, and netPanTro1) [42,43]. Similarly, a random list of promoter and cDNA sequences were obtained from Ensembl (http://www.ensembl.org) by referencing a genome coordinate list of all known human protein-encoding genes (version 35.1) with an Ensembl gene identifier [44]. For each gene, a coordinate range of length equal to the TFBS of interest was randomly picked in the (a) 3,500 bases 5′ to the gene start site and (b) within protein coding DNA sequence. Sites from these two lists of coordinates were randomly chosen to form a set of genome regions with the same number of members as each TFBS category: NRF2 (21), NFκB (21), p53 (83), p53 apoptosis (29), and p53 cell cycle/cell growth (23). This process was repeated 1,000 times with replacement to capture the variance in the data. As with the known TFBSs, the mammalian multiple alignment data from multiz17way were retrieved for each of these promoter and cDNA sites. The placement of target genes into p53 subcategories was based on the grouping used in Qian et al. and an annotated literature search (Table S1).

Pair-wise percent identities relative to the each TFBS consensus were calculated as the percentage of RE bases that were either (a) identical between the human and second genome or (b) mismatched but do not deviate from the consensus sequence (Figure 1). For p53 REs, the variable spacer region was not considered. The distribution of conservation for each TFBS set is shown in Figure 2. The conservation of the randomly chosen sets of coding region and promoter sequences, which represent the high and low extremes respectively of human genome conservation for TFBS comparisons, was calculated in the same fashion, and average results per x-axis bin for 1,000 trials are shown in Figure 2. *p*-Values describing the statistical difference between percent sequence identity means for each TFBS set (Table 4) were calculated using an unpaired two-tailed t-test with the assumption of unequal variances. The ORs in Table 4 were calculated as OR = ad/bc, where a = mean conservation of the column element, b = mean conservation of the row element, c = 100 − a, and d = 100 − b.

A list of CNC blocks, which represent the 5% most conserved portions of the human genome, were downloaded from the “most conserved” track of the UCSC genome browser. These were generated by constructing a phylogenetic two-state Hidden Markov Model [45] from the 17-way multiple alignment, which includes the human 35.1 genome release [31,41,44]. To generate the data in Table 5, the coordinates of each TFBS set were intersected with the CNC blocks. Likewise, the average number of randomly chosen promoter regions (1,000 trials) found within CNCs was determined. The probability of finding a greater fraction of CNCs in randomly chosen promoter regions over TFBS sites was calculated using a two-tailed binomial test.

For the ROC curves in Figure 3, the sensitivity (true positive rate) and 1-specificity (false positive rate) of TFBS prediction were calculated at each of 11 conservation thresholds. The true positive rate was the fraction of bona fide TFBSs with a consensus sequence percent identity above a given level. The false positive rate was calculated as the average number of sites (1,000 trials) that fell above a conservation threshold (consensus sequence percent identity) in an equivalently sized set of random promoter sequences. For example, 83 TFBSs composed the p53 ROC curve. Therefore, sets of 83 random promoter sites, where each site was 20 bases in length, were used to estimate the false positive rate. ROC AUCs (Table 6) and standard errors were calculated directly from the graphs using the trapezoid rule as described by Hanley and McNeil [46].

Positional sequence diversity for a TFBS, which is related to the inverse of sequence identity, was calculated as the percentage of human nucleotides (nt) at each position that had a nonconsensus mismatch or insertion/deletion event when compared to one of the four mammals (Figure 4). Each TFBS of a given class (i.e., TP53) and its alignment to a mammal (i.e., mouse) was pulled from the multiz multiple alignments to produce a set of paired sequence alignments. For each RE member of this pool, we counted the number of times where the first position of the mammalian sequence in the alignment differed from the human nt due to either (a) a nonconsensus mismatch or (b) an insertion/deletion event. This count was divided by the total number of TFBSs in a group (i.e., 83 for all p53 REs or 21 for all NFκB REs) to get the percentage of two-way alignments that differed at that position. This value is taken as the sequence diversity at that first position. This calculation was then performed for the remaining nts in the TFBS. Mismatches from human that did not alter the consensus motif did not increase the percent sequence diversity and were ignored.

## Supporting Information

Figure S1 Distribution of Human-to-Primate TFBS Conservation Scores (295 KB DOC)

Figure S2 ROC Curves for Four Human-to-Mammal Comparisons Indicate the Ability of Different Interspecific Comparisons to Identify Bona Fide TFBSs (332 KB DOC)

Figure S3 Alignments for 52 p53 REs across Seven Mammalian Species Obtained from UCSC Multi17way Alignment Tool (91 KB DOC)

Figure S4 Sequence Logos for Human p53 REs Used for Position Weight Matrix Model Construction (152 KB DOC)

Table S1 Validated TFBSs Used in This Study and Supplementary References (454 KB DOC)

Table S2 Coordinates of Validated TFBSs (152 KB DOC)

Table S3 Correlation between RE Identity Distributions and Distributions Obtained from Sampling of Promoter Fragments (39 KB DOC)

## Abbreviations

AUC: area under the curve
CNC: conserved noncoding region
NFκB: nuclear factor of kappa light chain gene enhancer in B cells
NRF2;: nuclear factor (erythroid-derived 2)-like 2 nuclear factor
OR: odds ratio
RE: response element
ROC: receiver operator characteristic
TFBS: transcription factor binding site
