# Supplementary material for: Divergent Evolution of Human p53 Binding Sites: Cell Cycle Versus Apoptosis
Source: PLoS Genet. 2007 Jul 27;3(7):e127. doi: 10.1371/journal.pgen.0030127 (PMC1934401; doi:10.1371/journal.pgen.0030127)
Supplement: Figure S1 — (295 KB DOC) [file pgen.0030127.sg001.doc]

Figure S1. Distribution of human to primate TFBS conservation scores. Human to chimpanzee (a) and human to rhesus monkey (*macaque*) (b) comparisons are graphed for each transcription factor as the percentage of all TFBSs from each gene group (TP53, 83 REs; NRF2, 21 REs; or NFKB, 21 REs) that fall within the x-axis conservation bins (red). Data for randomly chosen human genome promoter and coding region fragments (1000 trials, error bars are narrower than the line art) are shown in grey and blue, respectively. Averages and standard errors for the sets of known TFBSs are reported above each graph.

Figure S1
