# Supplementary material for: Divergent Evolution of Human p53 Binding Sites: Cell Cycle Versus Apoptosis
Source: PLoS Genet. 2007 Jul 27;3(7):e127. doi: 10.1371/journal.pgen.0030127 (PMC1934401; doi:10.1371/journal.pgen.0030127)
Supplement: Figure S2 — (332 KB DOC) [file pgen.0030127.sg002.doc]

Figure S2. ROC curves for four human to mammal comparisons (A-D) indicate the ability of different interspecific comparisons to identify bona fide transcription factor binding sites. Each data point on a curve represents a different conservation threshold to allow comparison in the indicated species. Using a dataset that is half experimentally verified TFBSs and half randomly chosen promoter sequences from human genes, the true positive and false positive prediction rates were calculated given conservation thresholds that classify a TFBS as authentic or spurious. The number of REs that compose each line are as follows: NRF2 (21), NFKB (21), TP53 (83), TP53 apoptotic (29), TP53 cell cycle/cell growth (23), TP53 with spacer of 0, 1, or 2 (i.e. the number of bona fide elements from table 1 with 2 or few bases between the half sites, 66), TP53 with spacer of 3 or more (i.e. the number of bona fide elements from Table 1 with 3 or more bases between the half sites, 17), most well known TP53 REs (30, designated in Table S1), and TP53 apoptotic REs compared to an alternative consensus sequence (29, using the consensus RNRCWWGNYN(N_0-13)_NRRCWWGYY).

Figure S2
