# Supplementary material for: Divergent Evolution of Human p53 Binding Sites: Cell Cycle Versus Apoptosis
Source: PLoS Genet. 2007 Jul 27;3(7):e127. doi: 10.1371/journal.pgen.0030127 (PMC1934401; doi:10.1371/journal.pgen.0030127)
Supplement: Figure S3 — (91 KB DOC) [file pgen.0030127.sg003.doc]

Figure S3: Alignments for 52 p53 REs across 7 mammalian species obtained from UCSC “Multi17way” alignment tool. Some gaps are created by insertions in species not shown. Spacer (n) between half sites is not considered in identity score. Mismatches (green) from human that do not alter the p53 consensus motif, RRRCWWGYYY(N_0-13_)RRRCWWGYYY, do not penalize the % sequence identity, whereas consensus-altering mismatches and insertion/deletion events do (red). For the p53 sites, spacer elements are in grey and are not considered when calculating percent identity. R=G or A; W=A or T; Y=C or T.

A. *CDKN1A -* cyclin-dependent kinase inhibitor (p21) (5’ element)

Consensus RRRCWWGYYYRRRCWWGYYY

human gaacatgtcccaacatgttg

chimp gaacatgtcccaacatgttg 100%

rhesus gaacatgtcccaacatgttg 100%

rat gaacatgtcttgacttgttc 95%

mouse gaacatgtcttgacatgttc 95%

rabbit gaacatgtcccaacatgtgt 95%

dog gaacatgtcccaacatgttg 100%

B. *PCNA* - proliferating cell nuclear antigen

Consensus RRRCWWGYYYRRR----CWWGYYY

human acatatgcccgga----cttgttc

chimp acatatgcccagc----cttgttc 95%

rhesus ------------------------ 0%

rat ------------------------ 0%

mouse ctcgcctcccagg----ctcctac 50%

rabbit ------------------------ 0%

dog acaa-–gcccaga----cttgttt 80%

C. *BBC3 -* BCL2 binding component 3 (*PUMA*)

Consensus RRRCWWGYYYRRRCWWGYYY

human ctgcaagtcctgacttgtcc

chimp -------------------- 0%

rhesus -------------------- 0%

rat -------------------- 0%

mouse ctgcaagccccgacttgtcc 95%

rabbit -------------------- 0%

dog -------------------- 0%

D. *BAX* - BCL2-associated X protein (3’ element)

Consensus RRRCWWGYYYRRRCWWGYYY

human agacaagcctgggcgtgggc

chimp agacaagcctgggcgtgggc 100%

rhesus agacaagcctgggcgtgggc 100%

rat -------------------- 0%

mouse -------------------- 0%

rabbit -------------------- 0%

dog -------------------- 0%

*E. CDKN1A -* cyclin-dependent kinase inhibitor (p21)

(3’ element)

Consensus RRRCWWGYYYRRRCWWGYYY

human gaagaagactgggcatgtct

chimp gaagaagactgggcatgtct 100%

rhesus gaagaagactgggcatgtct 100%

rat gaagaagcctgggcatgtct 100%

mouse gaagaagactgggcatgtct 100%

rabbit gaagccgaccgggcatgcct 90%

dog gaagaaaacggggcatgtct 90%

*F. CDKN1A -* cyclin-dependent kinase inhibitor (p21)

(Far 5’ element)

Consensus RRRCWWGYYYRRRCWWGYYY

human aaacatgccc(11)caacaagctg

chimp aaatatgccc----caacaagctg 95%

rhesus aaatatgcct----caacaagctg 95%

rat aaacatgcct----caacaaacca 90%

mouse aaatatgcct----caacaagcca 90%

rabbit aaacatgccc----caacaagcct 100%

dog aaatatgccc----caacaagcct 95%

*G. APAF1 –* apoptotic peptidase activating factor 1

Consensus RRRCWWGYYYRRRCWWGYYY

human agacatgtct(13)cgac-aagccc

chimp agacatgtct----cgac-aagccc 100%

rhesus agacatgtct----agttgaagccc 85.7%

rat agacatgtct----tttc-gaagcc 70%

mouse ggacatgtct----tctt-gaagcc 65%

rabbit ------------------------- 0%

dog cgacatgtct--------------- 45%

H. *BAX A* - BCL2-associated X protein

(5’ element)

Consensus RRRCWWGYYYRRRCWWGYYY

human tcacaagtt-a(1)agacaagcct

chimp tcacaagtt-a---agacaagcct 100%

rhesus tcacaagttaa---agacaagcct 95.2%

rat ------------------------ 0%

mouse ------------------------ 0%

rabbit ------------------------ 0%

dog ------------------------ 0%

I. *BID –* BH3 interacting domain death agonist

Consensus RRRCWWGYYYRRRCWWGYYY

human gggcatgatggtgcatgcct

chimp gggcatgatggtgcatgcct 100%

rhesus -------------------- 0%

rat -------------------- 0%

mouse -------------------- 0%

rabbit -------------------- 0%

dog -------------------- 0%

J. *CASP1* – caspase 1

Consensus RRRCWWGYYYRRRCWWGYYY

human agacatgcatatgcatgcac

chimp agacatgcatatgcatgcac 100%

rhesus agacatgcatatgcatgtgc 95%

rat ---catgcgcacacagtaat 60%

mouse ---catgcgcacacagcaat 60%

rabbit aggcatgcatatatatgttc 95%

dog -----tgtgtaggcaccaac 55%

K. *CTSD –* cathepsin D

Consensus RRRCWWGYYYRRRCWWGYYY

human aagct--gggccgggctg------------accc

chimp ---------------------------------- 0%

rhesus aagcg--gagccgggctg------------accc 90%

rat ---------------------------------- 0%

mouse aaagt--gggcgggcgcg------------ttcc 70%

rabbit agagcccggacaggcgcggcggcgggcgcgctcc 35.3%

dog ---------------------------------- 0%

L. *CASP6* – caspase 6

Consensus RRRCWWGYYYRRRCWWGYYY

human aggcaaggag(4)agacaagtct

chimp aggcaaggag---agacaagtct 100%

rhesus aggcaaggcg---agacaagtct 100%

rat ----------------------- 0%

mouse ----------------------- 0%

rabbit ---------------------ct 10%

dog ----------------------- 0%

M. *DCC1 –* defective in sister chromatid cohesion homolog 1

Consensus RRRCWWGYYYRRRCWWGYYY

human cagcatgttcacacaagcca

chimp gagcatgttcacaccagcca 95%

rhesus gagcatgttcacacaagcca 100%

rat atgcatacccctgctgcctg 65%

mouse gggtatgcccctggtgcctg 65%

rabbit gcgaatgttcacagaagcta 85%

dog aaaaaagtgcacagaagtgt 80%

N. *FAS* – TNF receptor superfamily, member 6

Consensus RRRCWWGYYYRRRCWWGYYY

human ggacaagccctgacaagcca

chimp ggacaagccctgacaagcca 100%

rhesus ggacaagccctgacaagcca 100%

rat -------------------- 0%

mouse agacttgcccagaca----- 75%

rabbit -------------------- 0%

dog ggaggggatcgagcc---cg 55%

O. *FOS –* v-fos FBJ murine osteosarcoma viral oncogene homolog

5’element

Consensus RRRCWWGYYYRRRCWWGYYY

human ggactt-gctc(1)gagc-gcacgc

chimp ggactt-gctc---gagc-gcacgc 100%

rhesus ggacttggctc---gagc-gcacgc 95.2%

rat ggactt-gtc----gagc-gcgtgc 95%

mouse ggactt-gtc----gagc-gcgtgc 95%

rabbit gggctt-gttg---gagc-gcacgc 95.2%

dog ggac-----tc---gtggggcgcac 61.9%

P. *FOS –* v-fos FBJ murine osteosarcoma viral oncogene homolog

3’element

Consensus RRRCWWGYYYRRRCWWGYYY

human acgc---ttgccatagtaagaat

chimp acgc---ttgccatagtaagaat 100%

rhesus aagc---ttgccatagtaagaat 100%

rat acac---ttgtcatactaagact 95%

mouse acac---ttgtcatagtaagact 100%

rabbit tcgccagtcgtcatagtaagcc- 73.9%

dog gcgc--gctgtcctagcgaggat 80.9%

Q. *IGFBP3 –* insulin-like growth factor binding protein 3

3’element

Consensus RRRCWWGYYYRRRCWWGYYY

human aaacaagcca(1)caacatgctt

chimp aaacaagcca---caacatgctt 100%

rhesus aaacaagcca---caacatgctt 100%

rat aaa-------------------- 15%

mouse aaataaactt---t--ctagcat 70%

rabbit ----------------------- 0%

dog ----------------------- 0%

R. *IGFBP3 –* insulin-like growth factor binding protein 3

5’element

Consensus RRRCWWGYYYRRRCWWGYYY

human gggcaa------gacctgccaagcct

chimp gggcaa------gacctgccaagcct 100%

rhesus gggcaa------gacctgccaagcct 100%

rat ggggag------aa------------ 25%

mouse ggggag------ta------------ 25%

rabbit -------------------------- 0%

dog cggcaggcactcggggcgcctggtcg 46.2%

S. *LRDD –* leucine-rich repeats and death domain containing

Consensus RRRCWWGYYYRRRCWWGYYY

human aggcctgcct(8)ggacatgtct

chimp aggcctgcct---ggacatgtct 100%

rhesus aggcctgcct---ggacatgtct 100%

rat aggcttgctt---ggacatgtct 100%

mouse aggcttgctt---ggacatgtct 100%

rabbit ----------------------- 0%

dog aggcctgttt---ggacatgtct 100%

T. *P53AIP1 –* p53-regulated apoptosis-inducing protein 1

Consensus RRRCWWGYYYRRRCWWGYYY

human tctcttgcccgggcttgtcg

chimp tttcttgcccgggcttgtcg 95%

rhesus tctcttgcccagacttgttg 100%

rat -------------------- 0%

mouse -------------------- 0%

rabbit -------------------- 0%

dog -------------------- 0%

U. *PCBP4 –* poly(rC) binding protein 4

Consensus RRRCWWGYYYRRRCWWGYYY

human gaacttaaga(10)ggacaagttg

chimp gaacttaaga----ggacaagttg 100%

rhesus gaacttaaga----ggacaagttg 100%

rat gagcttaaga----agaaaagtca 90%

mouse gatcttaagg----ggggaggtc- 75%

rabbit ------------------------ 0%

dog gagtctcagg----cgacaagttg 75%

V. *PERP –* TP53 apoptosis effector

Consensus RRRCWWGYYYRRRCWWGYYY

human a-----ggcaagctccagcttgttc

chimp ------------------------- 0%

rhesus a-----ggcaagctccagcttgttc 100%

rat gcctctggcaagctctgacttgtca 72%

mouse g-----ggcaagctctggcatgtca 90%

rabbit g-----gtagagttcaagtctgt-- 65%

dog a-----agcaggctcctacttgtca 85%

W. *PLAG1 –* pleiomorphic adenoma gene 1

Consensus RRRCWWGYYYRRRCWWGYYY

human caactagac----tagactagctt

chimp caactagac----tagactagctt 100%

rhesus caaccagacagactagactagctt 79.2%

rat ctgccggat----tcca-----tt 50%

mouse caactagac----tcca-----tg 60%

rabbit ------------------------ 0%

dog ------aac----taaactggctt 60%

X. *PMAIP1 –* phorbol-12-myristate-13-acetate-induced protein 1

Consensus RRRCWWGYYYRRRCWWGYYY

human gagcgtgtccgggcaggtcg

chimp gagcgtgtccgggcaggtcg 100%

rhesus gagcgtgtccgggcaggtcg 100%

rat cggcttgccccggcaagttg 90%

mouse aggcttgccccggcaagttg 95%

rabbit aggtccgggcaggcagggcg 70%

dog -------------------- 0%

Y. *SIVA –* SIVA1, apoptosis-inducing factor

5’ element

Consensus RRRCWWGYYYRRRCWWGYYY

human ctacatgcacacgcatgcat

chimp ctacatgcacacgcatgcat 100%

rhesus ctacttgcacacgcatgcat 100%

rat -gatatacacaagtatgca- 75%

mouse -------------------- 0%

rabbit -------------------- 0%

dog --acatgcaccc-------- 45%

Z. *SIVA –* SIVA1, apoptosis-inducing factor

3’ element

Consensus RRRCWWGYYYRRRCWWGYYY

human gtacttggcagggcatgtct

chimp gtacttggcagggcatgtct 100%

rhesus gtacttggcagggcatgtct 100%

rat -------------------- 0%

mouse -------------cctgtgt 25%

rabbit -------------------- 0%

dog -------------------- 0%

AA. *SIVA –* SIVA1, apoptosis-inducing factor

Far 5’ element

Consensus RRRCWWGYYYRRRCWWGYYY

human gcacaagcct(5)tgtctggagg

chimp gcacaagcct---tgtctggagg 100%

rhesus gcacaagcct---tgtctggagg 100%

rat gcacaagcct---tgtgaggact 95%

mouse gcacaagcct---tgtgaggagt 95%

rabbit gcacaagc-----tgtcaggact 90%

dog gcacaagcct---tgtt-ggagg 90%

AB. *TNFRSF10B –* tumor necrosis factor receptor superfamily, member 10b

Consensus RRRCWWGYYYRRRCWWGYYY

human cgtcttgcccggacatgccc

chimp cgtcttgcccggacatgccc 100%

rhesus cgtcttgcccggacatgccc 100%

rat -------------------- 0%

mouse -------------------- 0%

rabbit -------------------- 0%

dog -------------------- 0%

AC. *TNFRSF10C –* tumor necrosis factor receptor superfamily, member 10c

Consensus RRRCWWGYYYRRRCWWGYYY

human gggcatgtccgggcaggacg

chimp gggcatgtccgggcaggacg 100%

rhesus gggcatgtccaggcaggacg 100%

rat -------------------- 0%

mouse -------------------- 0%

rabbit -------------------- 0%

dog -------------------- 0%

AD. *TP53I3 –* tumor protein p53 inducible protein 3

Consensus RRRCWWGYYYRRRCWWGYYY

human cagcttgcccacccatgctc

chimp cagcttgcccacccatgctc 100%

rhesus cagcttgcccacccatactc 95%

rat -------------------- 0%

mouse -------------------- 0%

rabbit -------------------- 0%

dog -------------------- 0%

AE. *TP53INP1 –* tumor protein p53 inducible nuclear protein 1

Consensus RRRCWWGYYYRRRCWWGYYY

human gaacttgggggaacatgttt

chimp gaacttgggggaacatgttt 100%

rhesus gaacttgggagaacatgttt 95%

rat -------------------- 0%

mouse -------------------- 0%

rabbit -------------------- 0%

dog -------------------- 0%

AF. *TRAF4 –* TNF receptor-associated factor 4

Consensus RRRCWWGYYYRRRCWWGYYY

human gggcaagccagggcctgcct

chimp gggcaagccagggcctacct 95%

rhesus gggcaagccagggcctgcct 100%

rat gggcaagccagggcttgttt 100%

mouse gggcaagccagggcttgttt 100%

rabbit gggcaagcc----------c 50%

dog gggcaagcccagtcctgtct 95%

AG. *WIG1 –* zinc finger, matrin type 3

Consensus RRRCWWGYYYRRRCWWGYYY

human aaacaagtccagacatgcct

chimp aaacaagtccagacatgcct 100%

rhesus aaacaagtccagacatgcct 100%

rat aaacaagcccaggcatgtct 100%

mouse aaacaagcccaggcatgtct 100%

rabbit -------------------- 0%

dog aaacaagtccagacatgtct 100%

AH. *PTEN –* phosphatase and tensin homolog

Consensus RRRCWWGYYYRRRCWWGYYY

human gagcaagccc(14)gggcatgctc

chimp gagcaagccc----gggcatgctc 100%

rhesus gagcgagctc----gggcatgctc 95%

rat gagcgagcc-----gggcatgctc 90%

mouse gagcgagcc-----gggcatgctc 90%

rabbit gagcgagccc----gggcatgctc 95%

dog --------------gggcatg--- 35%

AI. *BTG2 –* BTG family, member 2

Consensus RRRCWWGYYYRRRCWWGYYY

human agtccgggca(1)agcccgag----ca

chimp agtccgggca---agcccgag----ca 100%

rhesus agtccgggca---agcccgag----ca 100%

rat agtccgggca---agcccgtg----ag 85%

mouse agtccgggca---agcccgag----ag 90%

rabbit --------------------------- 0%

dog agtccc-gca---ag-tcgagccctgc 62.5%

AJ. *DUSP1 –* dual specificity phosphatase 1

Consensus RRRCWWGYYYRRRCWWGYYY

human gaacttgtca(2)ggctttgttt

chimp gaacttgtca---ggctttgttt 100%

rhesus gaacttgtca---ggctttgttt 100%

rat gggcttgtca---ggctttgttt 100%

mouse gggcttgtca---ggctttgttt 100%

rabbit ggacttgtca---ggctttgtct 100%

dog ggacttgtcc---ggctttgttc 100%

AK. *TP73 –* tumor protein p73

Consensus RRRCWWGYYYRRRCWWGYYY

human gggcaagctgaggcctgccc

chimp -------------------- 0%

rhesus gggcaagctgaggcctgccc 100%

rat gggcaagctgaggcctgccc 100%

mouse gggcaagctgaggcctgccc 100%

rabbit -------------------- 0%

dog gggcaagccggggcctg-cc 95%

AL. *GDF15 –* growth differentiation factor 15

5’ element

Consensus RRRCWWGYYYRRRCWWGYYY

human catcttgcccagacttgtct

chimp catcttgcccagacttgtct 100%

rhesus cgtcttgcccagacttgtct 100%

rat -------------------- 0%

mouse -------------------- 0%

rabbit -------------------- 0%

dog -------------------- 0%

AM. *GDF15 –* growth differentiation factor 15

3’ element

Consensus RRRCWWGYYYRRRCWWGYYY

human agccatgcccgggcaagaac

chimp agccatgcccaggcaagaac 100%

rhesus agccatgccggggcaagaac 95%

rat ggccctgcgcgcgctccatg 70%

mouse ggccccgcccgcgctccagg 65%

rabbit -------------------- 0%

dog cggcatgcctggacagggac 85%

AN. *MDM2 –* transformed 3T3 cell double minute 2, p53 binding protein

5’ element

Consensus RRRCWWGYYYRRRCWWGYYY

human ggtcaagttcagacacgttc

chimp ggtcaagttcagacacgttc 100%

rhesus ggtcaagttcagacacgttc 100%

rat ggtcaagttaggacacgttc 95%

mouse ggtcaagttgggacacgtcc 95%

rabbit ggtcaagttcggacacattc 95%

dog ggacaagttcggactcctct 95%

AO. *MDM2 –* transformed 3T3 cell double minute 2, p53 binding protein3’ element

Consensus RRRCWWGYYYRRRCWWGYYY

human gagttaagtc(1)tgacttgtct

chimp gagttaagtc---tgacttgtct 100%

rhesus gagttaagtc---tgacttgtct 100%

rat gagctaagtc---tgacatgtct 100%

mouse gagctaagtc---tgacatgtct 100%

rabbit gagttaagtc---tgacttgtct 100%

dog gagctaagcc---cgacttgcct 95%

AP. *PLK2–* polo-like kinase 2

Far 5’ element

Consensus RRRCWWGYYYRRRCWWGYYY

human agaca------tggtg(3)aaactagctt

chimp agaca------tggtg---aaactagctt 100%

rhesus agaca------tggtg---aaactagctt 100%

rat gagca--------------aaactctctc 65%

mouse gagca--------------gaactctctc 65%

rabbit ----------------------------- 0%

dog aagtaaagggacggcg---ggac-ggctc 61.5%

AQ. *PLK2 –* polo-like kinase 2

5’ element

Consensus RRRCWWGYYYRRRCWWGYYY

human aaacatgcctggacttgccc

chimp aaacatgcctggacttgccc 100%

rhesus aaacatgcctggacttgccc 100%

rat caacatgcccgggcttgcag 85%

mouse caacatgcccgggcttgcat 90%

rabbit -------------------- 0%

dog aaacaggcctgggctcgccc 90%

AR. *PLK2 –* polo-like kinase 2

3’ element

Consensus RRRCWWGYYYRRRCWWGYYY

human ggtcatga—tt(3)taacttgcct

chimp ------------------------ 0%

rhesus ggtcatgg—tt---taacttgcct 95%

rat ggttattttct---tt-----cct 57.1%

mouse tgttcccgact---tttgtttcct 42.8%

rabbit ggtcgccgccc----------cct 42.8%

dog ------------------------ 0%

AS. *PLK3–* polo-like kinase 3

Consensus RRRCWWGYYYRRRCWWGYYY

human taacatgccc(6)aagcgagcgc

chimp taacatgccc---aagcgagcgc 100%

rhesus tgacacgcct---aagcgagcgc 95%

rat tgacacacac---aagtttgcaa 70%

mouse tgatgtgcac----agtttgcaa 65%

rabbit ----------------------- 0%

dog tgacgccccc---aggcgagctg 80%

AT. *SESN1 –* sestrin 1

Consensus RRRCWWGYYYRRRCWWGYYY

human ggac----------aagtctccacaagtca

chimp ggac----------aagtctccacaagtca 100%

rhesus ------------------------------ 0%

rat gtgc----------cggtgcgcaca----- 55%

mouse ctgc----------cggtgcgcacaagtca 75%

rabbit gtgc----------aagtctccgcaagccc 95%

dog gcacggcg(33)caaattctggccttgcca 28.1%

AU. *SNF –* SWI/SNF related, matrix associated, actin dependent regulator of chromatin, subfamily d, member 33’ element

Consensus RRRCWWGYYYRRRCWWGYYY

human tagcattagc(2)agacat---------------gtcc

chimp tagcattagc---agacat---------------gtcc 100%

rhesus tagcattagt---agacat---------------gtcc 100%

rat taaaattagc---gaaccctgaagtctctcgag-tccc 47.1

mouse taaaattagc---ggaccctgaagtctctcaagagccc 48.6%

rabbit --------gc---aaactt---------------gtcc 60%

dog tagtatctgc---aggcat---------------gctc 90%

AV. *EDN2–* endothelin 2

Consensus RRRCWWGYYYRRRCWWGYYY

human ctgcaagcccgggcatgccc

chimp ctgcaagcccgggcatgacc 95%

rhesus ctgcaagcccaggcatgccc 100%

rat ctgctagctgggaccaggcc 85%

mouse ctgcagattgggatcagacc 70%

rabbit ccgctagcctagacctgccc 90%

dog -------------------- 0%

AW. *SERTAD1 –* SERTA domain containing 1

Consensus RRRCWWGYYYRRRCWWGYYY

human gggcatgcgccctgaagcc---------c

chimp gggcatgcgccctgaagcc---------c 100%

rhesus gggcatgcgcccagaagcc---------c 100%

rat gggcatgcgctttagagacaggctccccc 51.7%

mouse gggcatgcgctttagggacaacctccccc 48.3%

rabbit gtgcctgcgtccttaagccaaactcggat 58.6%

dog aggcatgcgccctgaagccaccctcc--c 74.1%

AX. *TGFA –* transforming growth factor, alpha

Consensus RRRCWWGYYYRRRCWWGYYY

human ggg-caggccctgcctagtct

chimp ggg-caggccctgcctagtct 100%

rhesus ggg-caggcc-----tggtct 70%

rat ggg-ccggccctg--tcgcct 80%

mouse ggggccggccctg--tcgcct 76.2%

rabbit ggg-ccggccctgcctcgcca 85%

dog --------------------- 0%

AY. *DKK1–* dickkopf homolog 1

Consensus RRRCWWGYYYRRRCWWGYYY

human agccaagctt(6)aaccaagttc

chimp agccaagctt---aaccaagttc

rhesus aaccaagctt---aaccaagttc 100%

rat ----------------------- 0%

mouse ----------------------- 0%

rabbit agcaaagtct---aacaaagttt 90%

dog atccaaactc---agttaaattt 75%

AZ. *FLT1 –* fms-related tyrosine kinase 1

Consensus RRRCWWGYYYRRRCWWGYYY

human ggacacgctc(5)ggacctgagc

chimp ggacgcgctc---ggacctgagc 95%

rhesus ggacgcgctc---ggacctgagc 95%

rat agactccctc---ggacccgcgc 90%

mouse agactccctc---ggacccgcgc 90%

rabbit agacgcgctc---ggacccgagc 90%

dog agacgtgctc---ggacccgaga 85%

AAA. *NDRG1 –* N-myc downstream regulated gene 1

Consensus RRRCWWGYYYRRRCWWGYYY

human ccacatgcac(12)gcacatgaac

chimp ccacatgcac----gcacatgaac 100%

rhesus ccacatgcac----gcacatgaac 100%

rat ccgcaagcac----gcacgggaac 90%

mouse ccttatgcac----gcacgggcac 80%

rabbit ------------------------ 0%

dog -------cac----ac----ggac 40%
