# Supplementary material for: Divergent Evolution of Human p53 Binding Sites: Cell Cycle Versus Apoptosis
Source: PLoS Genet. 2007 Jul 27;3(7):e127. doi: 10.1371/journal.pgen.0030127 (PMC1934401; doi:10.1371/journal.pgen.0030127)
Supplement: Figure S4 — (152 KB DOC) [file pgen.0030127.sg004.doc]

**Figure S4. Sequence logos for human p53 response elements used for position weight matrix (PWM) model construction.** Sequence logo visualizes contributions of individual positions and individual nucleotides to the overall p53 binding motif. Generated by the WebLogo program ([http://weblogo.berkeley.edu](http://weblogo.berkeley.edu/)). The height of each letter is proportional to its frequency in the position weight matrix, and the height of the entire stack is adjusted to signify the ‘information content’ (measured in bits) of the sequences at that position. The logo displays both significant residues and subtle sequence patterns.

**Figure S4**

A. Logo for 83 human p53 response elements

B. Logo for 23 cell-cycle related human p53 response elements

C. Logo for 29 apoptosis related human p53 response elements
