# Supplementary material for: Divergent Evolution of Human p53 Binding Sites: Cell Cycle Versus Apoptosis
Source: PLoS Genet. 2007 Jul 27;3(7):e127. doi: 10.1371/journal.pgen.0030127 (PMC1934401; doi:10.1371/journal.pgen.0030127)
Supplement: Table S1 — (454 KB DOC) [file pgen.0030127.st001.doc]

**Table S1: Validated TFBSs used in this study.**

| **Target gene** | **Experimental support** |
| --- | --- |
| **TP53 binding sites** | |
| ***ADARB1*** | *ADARB1* was upregulated in a TP53-dependent manner after UV exposure, and RE functionality was confirmed using yeast reporter assays as well as luciferase reporter assays in human cells [1]. |
| ***APAF1*** | *APAF1* expression is induced in a TP53-dependent fashion following exposure to camptothecin in human neurons, and the functional RE was identified using EMSA and luciferase assays [2]. |
| ***ARHGEF7*** | *ARHGEF7*  was upregulated in a TP53-dependent manner after UV exposure, and RE functionality was confirmed using yeast reporter assays as well as luciferase reporter assays in human cells [1]. |
| ***ATF3*** | *ATF3* was induced in a TP53-dependent manner following exposure to a variety of stresses in human cells. The RE was confirmed using EMSA and luciferase assays in human osteosarcoma cell lines [3]. The RE region was physically associated with TP53 in chromatin immunoprecipitation assays [4]. |
| ***BAX*** | The RE region was physically associated with TP53 in chromatin immunoprecipitation assays [4]. RE function was confirmed using luciferase assays in human cell lines using both wild-type and mutant versions of TP53 [5]. |
| ***BBC3*** | *BBC3* is induced following TP53 activation, and the functional RE was verified by EMSA assays [6-8]. The RE region was also physically associated with TP53 in chromatin immunoprecipitation assays [4]. |
| ***BID*** | *BID* is induced in a TP53-dependent manner in irradiated mice and the functional RE was identified in human cells using EMSA, luciferase, and chromatin immunoprecipitation assays [9]. |
| ***BTG2*** | *BTG2*’s antiproliferative activity was confirmed using transformation assays in NIH3T3 cells. BTG2 induction was observed in a TP53-dependent manner following irradiation. RE function was verified using β-galactosidase reporter [10] and chromatin immunoprecipitation assays [4]. |
| ***C12ORF5*** | *C12ORF5* was expressed in a TP53-dependent manner after exposure to ionizing radiation and the RE region was recovered in quantitative chromatin immunoprecipitation assay in human cells. The functional RE was identified informatically and verified using a luciferase reporter construct. [4] |
| ***CASP1*** | mRNA levels were increased upon overexpression of TP53 in human breast cancer cells. The functional RE was verified using EMSA and β−galactosidase reporter assays [11]. |
| ***CASP6*** | Expression was induced in a TP53-dependent manner after adriamycin exposure. The specific RE was identified using EMSA and luciferase reporter assays in a variety of human cell lines [12]. |
| ***CDKN1A*** | *CDKN1A*, also known as *p21*, is the best characterized TP53-regulated gene with two extensively tested REs [13]. Regions housing several REs were recovered in a quantitative chromatin immunoprecipitation assay after exposure to ionizing radiation [4] as well as adriamycin [14,15]. A third, new site was recently identified using chromatin immunoprecipitation and luciferase assays in human cells [16]. |
| ***CTSD*** | Expression was induced in a TP53-dependent manner and RE functionality was confirmed using a luciferase reporter assay in human cell lines [17]. |
| ***DCC1*** | *DCC1* was upregulated after UV exposure, and RE functionality was confirmed using yeast reporter assays as well as luciferase reporter assays in human cells [1]. |
| ***DDB2*** | Expression was induced in a TP53-dependent manner after adriamycin treatment in human epithelial cells, and the RE was verified by quantitative chromatin immunoprecipitation assays [4,14]. |
| ***DDIT4*** | Expression was induced in a TP53-dependent manner following DNA damage in a variety of tissues and the responsible RE was confirmed using a luciferase reporter system [18]. |
| ***DKK1*** | Expression was upregulated upon TP53 induction and luciferase reporter assays confirms RE functionality [19]. |
| ***DSC3*** | *DSC3* has a CpG island promoter that contains a TP53-binding site confirmed by both EMSA and quantitative chromatin immunoprecipitation assays [20]. |
| ***DUSP1*** | Expression was upregulated in the human GM cell system, which contains endogenous nonfunctional TP53 and exogenous TP53 inducible from a Dex-driven promoter. The specific RE was verified using EMSA and luciferase reporter assays in human cells. [21] |
| ***DUSP5*** | Expression was dramatically increased upon addition of exogenous TP53 in a TP53-mutant human cell line. The RE region was identified by chromatin immunoprecipitation and confirmed using luciferase reporter assays in human cells. [22] |
| ***EDN2*** | Expression was induced in a TP53-dependent manner after adriamycin treatment in human epithelial cells, and the RE was verified by quantitative chromatin immunoprecipitation assays [14]. |
| ***EOMES*** | Expression was upregulated following UV exposure, and RE functionality was confirmed using yeast reporter assays as well as luciferase reporter assays in human cells [1]. |
| ***FAS*** | *FAS* is TP53-inducible after a variety of exposures in human cells, and the functional RE was verified using luciferase reporter assays [23]. The RE region was physically associated with TP53 in chromatin immunoprecipitation assays as well [4]. |
| ***FDXR*** | Expression was induced in a TP53-dependent fashion in numerous human cell lines and the functional RE was identified using luciferase reporter assays [24,25]. Additionally, the RE region was physically associated with TP53 in chromatin immunoprecipitation assays [4]. |
| ***FLT1*** | Expression was induced in a TP53-dependent manner and the functional RE was verified using luciferase reporter assays in human cells [26]. |
| ***FOS*** | Expression was TP53-inducible and two contiguous functional REs were verified using EMSA, luciferase, and yeast transactivation assays [27,28], |
| ***GADD45A*** | The RE region was physically associated with TP53 in chromatin immunoprecipitation assays [4]. RE function was confirmed using luciferase assays in human Saos-2 cells using both wild-type and mutant versions of TP53 [5]. |
| ***GDF15*** | Two REs, one which acts as an enhancer and the other as an expression silencer, have been identified and confirmed using EMSA and luciferase reporter assays in human cells [29]. RE region was physically associated with TP53 in chromatin immunoprecipitation assays [4]. |
| ***GPX1*** | The RE region was recovered following chromatin immunoprecipitation assays in human cells after TP53 induction by ionizing radiation. The RE was identified informatically and verified using a luciferase reporter construct. [4] |
| ***IGFBP3*** | Expressed in a TP53-inducible manner in human cells and the function of two distinct REs was verified using EMSA and luciferase reporter assays [30]. |
| ***LRDD*** | *LRDD* suppresses tumor cell growth and induces apoptosis in a TP53-dependent fashion. The responsible RE was identified using luciferase reporter assays [31]. |
| ***MASPIN1*** | *MASPIN1* has a CpG island promoter that contains 2 TP53-binding sites confirmed by chromatin immunoprecipitation assays. [20] The functionality of both sites were confirmed using a luciferase reporter construct. [32] |
| ***MDM2*** | Expression was induced in a TP53-dependent manner after adriamycin treatment in human epithelial cells, and the RE verified by chromatin immunoprecipitation assays [14,15]. The functionality of two distinct REs was confirmed using McKay and luciferase assays in human cells [5,33]. |
| ***MLH1*** | Expression was induced in a TP53-dependent manner in response to DNA damaging agents. The functional RE was identified by chromatin precipitation and luciferase reporter assays in human cells. [34] |
| ***MMP2*** | Expressed in a TP53-inducible manner in human cells and the RE was verified using EMSA and luciferase reporter assays [35]. |
| ***NDRG1*** | Expression was induced by DNA damage in a TP53-dependent fashion. Luciferase assays confirm the function of the RE. [36] |
| ***P53AIP1*** | Expression was induced in a TP53-dependent manner and leads to cell death. The functional RE was verified using EMSA and luciferase reporter assays in human cells [37]. The RE region was physically associated with TP53 in chromatin immunoprecipitation assays [4]. |
| ***PCBP4*** | Expression was induced by DNA damage in a TP53-dependent fashion. EMSA and luciferase assays in human cell lines confirm the function of the RE. [38] |
| ***PCNA*** | Expression was induced in a TP53-dependent manner and the RE was verified in yeast transactivation assays and a CAT activity reporter system in human cells [27,39]. |
| ***PDGFC*** | Expression was induced in a TP53-dependent manner after adriamycin treatment in human epithelial cells, and the RE was verified by chromatin immunoprecipitation assays. [14] |
| ***PERP*** | Two sites were identified as required for TP53 induction using EMSA, chromatin immunoprecipitation, and luciferase reporter assays in human cells [40]. However, we could locate only one site, site ‘D’, in the May 2004 human genome release. |
| ***PHLDA3*** | The RE was recovered by chromatin immunoprecipitation in human cells following *TP53* induction by ionizing radiation. The RE was further verified using a luciferase reporter construct [4]. |
| ***PLAGL1*** | Expression was induced in a TP53-dependent manner and RE functionality was confirmed using a luciferase reporter assay in human cell lines [41]. |
| ***PLK2*** | Expression was induced following ionizing radiation in a TP53-dependent manner. Luciferase reporter assays in human cells confirmed the independent functionality of three distinct TP53 REs [42]. |
| ***PLK3*** | The RE was recovered by chromatin immunoprecipitation in human cells following *TP53* induction by ionizing radiation. The RE was further verified using a luciferase reporter construct [4]. |
| ***PMAIP1*** | The RE region was physically associated with TP53 in chromatin immunoprecipitation assays [4], and functionality was confirmed by yeast transactivation assays [27]. |
| ***PMS2*** | Expression was induced in a TP53-dependent manner in response to DNA damage. The functional RE was identified by chromatin immunoprecipitation and luciferase reporter assays in human cells. [34] |
| ***PPM1J*** | Expression was induced in a TP53-dependent manner after adriamycin treatment in human epithelial cells, and the RE was verified by chromatin immunoprecipitation assays. [14] |
| ***PRKAB1*** | The RE was recovered by chromatin immunoprecipitation in human cells following *TP53* induction by ionizing radiation. The RE was further verified using a luciferase reporter construct [4]. |
| ***PTEN*** | Expressed in a TP53-inducible manner in human cells and the RE was verified using EMSA and luciferase reporter assays [43]. |
| ***RPS27L*** | Expression was induced in a TP53-dependent manner after adriamycin treatment in human epithelial cells, and the RE verified by chromatin immunoprecipitation assays. [14] |
| ***RRM2B*** | Expression was induced by UV and γ-radiation in a TP53-dependent fashion. EMSA and luciferase reporter assays in human cells verified the function of the RE [44]. |
| ***SCARA3*** | Gene expression is elevated in a TP53-dependent manner in response to oxidative stress [45]. Confirmed RE functionality using a yeast-based transactivation assay [46]. |
| ***SCGB1D2*** | Expression was upregulated after UV exposure, and RE functionality was confirmed using yeast reporter assays as well as luciferase reporter assays in human cells [1]. |
| ***SEMA3B*** | Expressed in a TP53-inducible manner in human cells. RE was verified using EMSA and luciferase reporter assays [47]. |
| ***SERPINE1*** | Expressed in a TP53-inducible manner, and RE functionality was confirmed using EMSA assays and DNase I footprinting [48]. |
| ***SERTAD1*** | Expression was upregulated after UV exposure and RE functionality was confirmed using yeast reporter assays as well as luciferase reporter assays in human cells [1]. |
| ***SESN1*** | This gene and members of its family are regulated by TP53 [49], and RE functionality was confirmed using EMSA and luciferase reporter assays [50]. |
| ***SFN*** | Expression was induced in a TP53-dependent manner [51], and RE function was confirmed using luciferase assays in human cells using both wild-type and mutant versions of TP53 [5]. |
| ***SIVA*** | Expression was regulated by TP53 and sufficient to induce neuronal cell death. The function of three distinct REs was verified using EMSA and β−galactosidase reporter assays [52]. |
| ***SOD2*** | Expression was upregulated in a TP53-dependent manner upon doxorubicin exposure and RE function was verified using β−galactosidase reporter assays [53]. |
| ***TGFA*** | TP53 induction leads to three-fold accumulation of *TGFA* mRNA. The functional TP53 RE was identified using EMSA, cotransfection, and luciferase reporter assays in human cells [54]. |
| ***TNFRSF10B*** | Expression was induced in a TP53-dependent fashion following a variety of exposures [55]. An intronic RE was identified using luciferase reporter assays [56], EMSA [57], and chromatin immunoprecipitation assays [4]. |
| ***TNFRSF10C*** | Expression was TP53-inducible in gastrointestinal tract tumors [58] and the functional RE was verified using luciferase reporter assays in human cells [59]. |
| ***TP53I3*** | Expression was induced in a TP53-dependent fashion and the functional RE was identified using luciferase reporter assays [60]. The RE region was recovered during chromatin immunoprecipitation experiments following exposure to adriamycin or ionizing radiation [15]. |
| ***TP53INP1*** | Expression was TP53-inducible and the responsible binding site was confirmed using EMSA and luciferase reporter assays [61]. |
| ***TP73*** | Expression was induced in a TP53-dependent manner, and the functional RE was identified using luciferase reporter assays in human cells [62]. |
| ***TRAF4*** | Expression was induced in a TP53-dependent manner in response to gamma radiation. The functional TP53 RE was identified using EMSA, cotransfection, and luciferase reporter assays [63]. |
| ***UBTD1*** | Expression was induced in a TP53-dependent manner after adriamycin treatment in human epithelial cells, and the RE verified by chromatin immunoprecipitation assays. [14] |
| ***WIG1*** | Expression was induced in a TP53-dependent manner and overexpression leads to apoptosis [64]. The RE region was pulled down in a quantitative chromatin immunoprecipitation assay in human cells after exposure to ionizing radiation [4]. |
| ***XRCC5*** | Expression was induced by DNA damaging agents in a TP53-dependent manner and the functional RE was identified by chromatin immunoprecipitation assays. [65] |
| **NRF2 binding sites** | |
| ***ABCC1*** | An ARE element that acts as a transcriptional enhancer was identified using DNase I footprinting and gel mobility shift assays [66]. |
| ***ETS1*** | Hydrogen peroxide increases *ETS1* expression, and an ARE has been identified and functionally confirmed using luciferase reporter assays [67]. |
| ***FTH1*** | Expression was induced by pro-oxidants and the function of two distinct ARE elements was verified using mutational analysis and chromatin immunoprecipitation assays [68]. |
| ***FTL*** | Expression was induced by antioxidants and the responsible ARE was verified using luciferase reporter assays [69]. |
| ***GCLC*** | Expression was induced by beta-naphthoflavone and the functional ARE was verified using luciferase reporter assays [70]. |
| ***GCLM*** | Expression was induced by beta-naphthoflavone and two functional AREs were verified using luciferase reporter assays [71]. |
| ***GNAI2*** | Expression was induced by pro-oxidants and ARE function was verified using both mutational analysis of the binding site and EMSA assays [72]. |
| ***GPX2*** | Expression was induced by pro-oxidants and the function of two distinct ARE elements was verified using reporter gene and chromatin immunoprecipitation assays [73]. |
| ***GSTP1*** | Expression was induced by pro-oxidants and the functional ARE element was verified using EMSA and luciferase reporter gene assays [74]. |
| ***HBB*** | Expression was induced by pro-oxidants and the functional ARE element was verified using DNase I footprinting, EMSA, and chromatin immunoprecipitation assays [75]. |
| ***HMBS*** | Expression was induced by pro-oxidants and the functional ARE was verified using both mutational analysis of the binding site and UV crosslinking assays [76]. |
| ***NQO1*** | Expression was induced by beta-naphthoflavone and the functional ARE was verified using mutational analysis and EMSA assays [77]. |
| ***S100A6*** | Expression was increased by agents inducing oxidative stress and the functional ARE was verified using luciferase reporter assays [78]. |
| ***SAT*** | Expression was increased in response to cell treatment with a class of antineoplastic polyamine analogues. The functional ARE was verified using EMSA and luciferase reporter assays [79]. |
| ***SPTA1*** | The functional RE was identified using EMSA assays and DNase I footprinting analysis [80]. |
| ***TBXAS1*** | The functional RE was identified using mutational analysis, EMSA, and chromatin immunoprecipitation assays [81]. |
| ***TXNRD1*** | Expression was induced by a variety of oxidants and the functionality of two distinct REs was confirmed by EMSA and luciferase reporter assays [81]. |
| ***UGT1A6*** | Expression was induced by a variety of antioxidants and the functionality of two distinct REs was confirmed by transfection of mutated promoter constructs as well as EMSA assays [82]. |
| **NFKB binding sites** | |
| ***ALOX12*** | The functional NFKB RE was identified using luciferase reporter assays in human cells [83]. |
| ***CCL20*** | NFKB inducibility was demonstrated and the functional NFKB RE was identified using EMSA and luciferase reporter assays in human cells [84]. |
| ***CXCL1*** | NFKB inducibility was demonstrated and the functional NFKB RE was identified using EMSA, chromatin immunoprecipitation, and UV crosslinking assays in human cells [85,86]. |
| ***ELK1*** | Expression was induced by NFKB and the functional RE was identified using EMSA and luciferase assays in human cells [87]. |
| ***F8*** | Expression was NFKB inducible and the functional RE was pinpointed using EMSA, luciferase assays, and DNase I footprinting [88]. |
| ***ICAM1*** | A combination of mutagenesis experiments and luciferase and EMSA assays revealed that a well-characterized TNF-alpha regulatory site also has an NFKB responsive RE [89]. |
| ***IL6*** | Expression was NFKB inducible and the functional RE was pinpointed using EMSA and luciferase assays in human cells [90]. |
| ***IL8*** | Expression was NFKB inducible and the functional RE was pinpointed using EMSA and luciferase assays in human cells [91]. |
| ***IRF1*** | Expression was NFKB inducible and the functional RE was pinpointed using both EMSA assays in human cells in a variety of cell lines [92,93] and in vivo DNase I footprinting [94]. |
| ***LTA*** | Expression was NFKB inducible and the functional RE was pinpointed using mutational analysis of the *LTA* promoter coupled with transactivation ability of these mutated constructs [95]. |
| ***MAT2A*** | Expression was NFKB inducible and the functional RE was pinpointed using EMSA, luciferase assays, and DNase I footprinting analysis in human cells [96]. |
| ***NFKBIA*** | The functional NFKB RE was identified using EMSA and luciferase reporter assays in human cells [57]. |
| ***PTGS2*** | Expression was induced in a NFKB-dependent manner [86] and a functional RE was identified using luciferase reporter assays [97]. |
| ***SDC4*** | Expression was induced in a NFKB-dependent manner and the binding site was verified using a luciferase reporter assay [86,98]. |
| ***SELE*** | Expression was NFKB inducible and the functional RE was pinpointed using mutational and transactivation analysis of *SELE*  promoter constructs as well as EMSA assays [99]. |
| ***SERPINE1*** | NFKB inducibility was demonstrated and the functional NFKB RE was identified using luciferase assays [100]. |
| ***TNIP1*** | Expression was induced in an NFKB-dependent manner and the functional RE was verified using informatics queries, luciferase reporter gene assays, and chromatin immunoprecipitation experiments [86]. |
| ***TNFRSF10B*** | Expression was induced in a NFKB-dependent manner and the functional RE was verified using EMSA, luciferase, and chromatin immunoprecipitation assays in human cells [57]. |
| ***TP53*** | NFKB inducibility was demonstrated and the functional NFKB RE was identified using EMSA assays and deletion analysis of promoter constructs containing the binding site [101]. |
| ***TRAF1*** | Expression was induced by NFKB and the functional binding site was identified using *in vitro* DNA binding assays, promoter-reporter gene assays, and RNase protection assays [86,102]. |
| ***ZFP36*** | This gene was induced in a NFKB-dependent manner [86], and an intron fragment that was required for expression of the *ZFP36* houses a NFKB binding site that was probed using EMSA assays [103]. |

^1^ TP53 consensus sequence = RRRCWWGYYY(N_0-13_)RRRCWWGYYY. The number of spacer bases are shown in parentheses.

^2^ NRF2 consensus sequence = TMAnnRTGAYnnnGCRWWW

^3^ NFKB consensus sequence = GGGRnnYY

**Supplementary References**

1. Tomso DJ, Inga A, Menendez D, Pittman GS, Campbell MR, et al. (2005) Functionally distinct polymorphic sequences in the human genome that are targets for TP53 transactivation. Proc Natl Acad Sci U S A 102: 6431-6436.

2. Fortin A, Cregan SP, MacLaurin JG, Kushwaha N, Hickman ES, et al. (2001) APAF1 is a key transcriptional target for TP53 in the regulation of neuronal cell death. J Cell Biol 155: 207-216.

3. Zhang C, Gao C, Kawauchi J, Hashimoto Y, Tsuchida N, et al. (2002) Transcriptional activation of the human stress-inducible transcriptional repressor ATF3 gene promoter by TP53. Biochem Biophys Res Commun 297: 1302-1310.

4. Jen KY, Cheung VG (2005) Identification of novel TP53 target genes in ionizing radiation response. Cancer Res 65: 7666-7673.

5. Kato S, Han SY, Liu W, Otsuka K, Shibata H, et al. (2003) Understanding the function-structure and function-mutation relationships of TP53 tumor suppressor protein by high-resolution missense mutation analysis. Proc Natl Acad Sci U S A 100: 8424-8429.

6. Nakano K, Vousden KH (2001) PUMA, a novel proapoptotic gene, is induced by TP53. Mol Cell 7: 683-694.

7. Yu J, Wang Z, Kinzler KW, Vogelstein B, Zhang L (2003) PUMA mediates the apoptotic response to TP53 in colorectal cancer cells. Proc Natl Acad Sci U S A 100: 1931-1936.

8. Jeffers JR, Parganas E, Lee Y, Yang C, Wang J, et al. (2003) Puma is an essential mediator of TP53-dependent and -independent apoptotic pathways. Cancer Cell 4: 321-328.

9. Sax JK, Fei P, Murphy ME, Bernhard E, Korsmeyer SJ, et al. (2002) BID regulation by TP53 contributes to chemosensitivity. Nat Cell Biol 4: 842-849.

10. Rouault JP, Falette N, Guehenneux F, Guillot C, Rimokh R, et al. (1996) Identification of BTG2, an antiproliferative TP53-dependent component of the DNA damage cellular response pathway. Nat Genet 14: 482-486.

11. Gupta S, Radha V, Furukawa Y, Swarup G (2001) Direct transcriptional activation of human caspase-1 by tumor suppressor TP53. J Biol Chem 276: 10585-10588.

12. MacLachlan TK, El-Deiry WS (2002) Apoptotic threshold is lowered by TP53 transactivation of caspase-6. Proc Natl Acad Sci U S A 99: 9492-9497.

13. Espinosa JM, Emerson BM (2001) Transcriptional regulation by TP53 through intrinsic DNA/chromatin binding and site-directed cofactor recruitment. Mol Cell 8: 57-69.

14. Hearnes JM, Mays DJ, Schavolt KL, Tang L, Jiang X, et al. (2005) Chromatin immunoprecipitation-based screen to identify functional genomic binding sites for sequence-specific transactivators. Mol Cell Biol 25: 10148-10158.

15. Szak ST, Mays D, Pietenpol JA (2001) Kinetics of TP53 binding to promoter sites in vivo. Mol Cell Biol 21: 3375-3386.

16. Saramaki A, Banwell CM, Campbell MJ, Carlberg C (2006) Regulation of the human p21(waf1/cip1) gene promoter via multiple binding sites for TP53 and the vitamin D3 receptor. Nucleic Acids Res 34: 543-554.

17. Wu GS, Saftig P, Peters C, El-Deiry WS (1998) Potential role for cathepsin D in TP53-dependent tumor suppression and chemosensitivity. Oncogene 16: 2177-2183.

18. Ellisen LW, Ramsayer KD, Johannessen CM, Yang A, Beppu H, et al. (2002) REDD1, a developmentally regulated transcriptional target of p63 and TP53, links p63 to regulation of reactive oxygen species. Mol Cell 10: 995-1005.

19. Wang J, Shou J, Chen X (2000) Dickkopf-1, an inhibitor of the Wnt signaling pathway, is induced by TP53. Oncogene 19: 1843-1848.

20. Oshiro MM, Watts GS, Wozniak RJ, Junk DJ, Munoz-Rodriguez JL, et al. (2003) Mutant TP53 and aberrant cytosine methylation cooperate to silence gene expression. Oncogene 22: 3624-3634.

21. Li M, Zhou JY, Ge Y, Matherly LH, Wu GS (2003) The phosphatase MKP1 is a transcriptional target of TP53 involved in cell cycle regulation. J Biol Chem 278: 41059-41068.

22. Ueda K, Arakawa H, Nakamura Y (2003) Dual-specificity phosphatase 5 (DUSP5) as a direct transcriptional target of tumor suppressor TP53. Oncogene 22: 5586-5591.

23. Muller M, Wilder S, Bannasch D, Israeli D, Lehlbach K, et al. (1998) TP53 activates the CD95 (APO-1/Fas) gene in response to DNA damage by anticancer drugs. J Exp Med 188: 2033-2045.

24. Liu G, Chen X (2002) The ferredoxin reductase gene is regulated by the TP53 family and sensitizes cells to oxidative stress-induced apoptosis. Oncogene 21: 7195-7204.

25. Hwang PM, Bunz F, Yu J, Rago C, Chan TA, et al. (2001) Ferredoxin reductase affects TP53-dependent, 5-fluorouracil-induced apoptosis in colorectal cancer cells. Nat Med 7: 1111-1117.

26. Menendez D, Krysiak O, Inga A, Krysiak B, Resnick MA, et al. (2006) A SNP in the flt-1 promoter integrates the VEGF system into the TP53 transcriptional network. Proc Natl Acad Sci U S A 103: 1406-1411.

27. Inga A, Storici F, Darden TA, Resnick MA (2002) Differential transactivation by the TP53 transcription factor is highly dependent on TP53 level and promoter target sequence. Mol Cell Biol 22: 8612-8625.

28. Elkeles A, Juven-Gershon T, Israeli D, Wilder S, Zalcenstein A, et al. (1999) The c-fos proto-oncogene is a target for transactivation by the TP53 tumor suppressor. Mol Cell Biol 19: 2594-2600.

29. Wong J, Li PX, Klamut HJ (2002) A novel TP53 transcriptional repressor element (TP53TRE) and the asymmetrical contribution of two TP53 binding sites modulate the response of the placental transforming growth factor-beta promoter to TP53. J Biol Chem 277: 26699-26707.

30. Buckbinder L, Talbott R, Velasco-Miguel S, Takenaka I, Faha B, et al. (1995) Induction of the growth inhibitor IGF-binding protein 3 by TP53. Nature 377: 646-649.

31. Lin Y, Ma W, Benchimol S (2000) Pidd, a new death-domain-containing protein, is induced by TP53 and promotes apoptosis. Nat Genet 26: 122-127.

32. Zou Z, Gao C, Nagaich AK, Connell T, Saito S, et al. (2000) TP53 regulates the expression of the tumor suppressor gene maspin. J Biol Chem 275: 6051-6054.

33. Zauberman A, Flusberg D, Haupt Y, Barak Y, Oren M (1995) A functional TP53-responsive intronic promoter is contained within the human mdm2 gene. Nucleic Acids Res 23: 2584-2592.

34. Chen J, Sadowski I (2005) Identification of the mismatch repair genes PMS2 and MLH1 as TP53 target genes by using serial analysis of binding elements. Proc Natl Acad Sci U S A 102: 4813-4818.

35. Bian J, Sun Y (1997) Transcriptional activation by TP53 of the human type IV collagenase (gelatinase A or matrix metalloproteinase 2) promoter. Mol Cell Biol 17: 6330-6338.

36. Stein S, Thomas EK, Herzog B, Westfall MD, Rocheleau JV, et al. (2004) NDRG1 is necessary for TP53-dependent apoptosis. J Biol Chem 279: 48930-48940.

37. Oda K, Arakawa H, Tanaka T, Matsuda K, Tanikawa C, et al. (2000) TP53AIP1, a potential mediator of TP53-dependent apoptosis, and its regulation by Ser-46-phosphorylated TP53. Cell 102: 849-862.

38. Zhu J, Chen X (2000) MCG10, a novel TP53 target gene that encodes a KH domain RNA-binding protein, is capable of inducing apoptosis and cell cycle arrest in G(2)-M. Mol Cell Biol 20: 5602-5618.

39. Morris GF, Bischoff JR, Mathews MB (1996) Transcriptional activation of the human proliferating-cell nuclear antigen promoter by TP53. Proc Natl Acad Sci U S A 93: 895-899.

40. Reczek EE, Flores ER, Tsay AS, Attardi LD, Jacks T (2003) Multiple response elements and differential TP53 binding control Perp expression during apoptosis. Mol Cancer Res 1: 1048-1057.

41. Rozenfeld-Granot G, Krishnamurthy J, Kannan K, Toren A, Amariglio N, et al. (2002) A positive feedback mechanism in the transcriptional activation of Apaf-1 by TP53 and the coactivator Zac-1. Oncogene 21: 1469-1476.

42. Burns TF, Fei P, Scata KA, Dicker DT, El-Deiry WS (2003) Silencing of the novel TP53 target gene Snk/Plk2 leads to mitotic catastrophe in paclitaxel (taxol)-exposed cells. Mol Cell Biol 23: 5556-5571.

43. Stambolic V, MacPherson D, Sas D, Lin Y, Snow B, et al. (2001) Regulation of PTEN transcription by TP53. Mol Cell 8: 317-325.

44. Tanaka H, Arakawa H, Yamaguchi T, Shiraishi K, Fukuda S, et al. (2000) A ribonucleotide reductase gene involved in a TP53-dependent cell-cycle checkpoint for DNA damage. Nature 404: 42-49.

45. Nakamura Y (2004) Isolation of TP53-target genes and their functional analysis. Cancer Sci 95: 7-11.

46. Tokino T, Thiagalingam S, el-Deiry WS, Waldman T, Kinzler KW, et al. (1994) TP53 tagged sites from human genomic DNA. Hum Mol Genet 3: 1537-1542.

47. Ochi K, Mori T, Toyama Y, Nakamura Y, Arakawa H (2002) Identification of semaphorin3B as a direct target of TP53. Neoplasia 4: 82-87.

48. Kunz C, Pebler S, Otte J, von der Ahe D (1995) Differential regulation of plasminogen activator and inhibitor gene transcription by the tumor suppressor TP53. Nucleic Acids Res 23: 3710-3717.

49. Budanov AV, Sablina AA, Feinstein E, Koonin EV, Chumakov PM (2004) Regeneration of peroxiredoxins by TP53-regulated sestrins, homologs of bacterial AhpD. Science 304: 596-600.

50. Velasco-Miguel S, Buckbinder L, Jean P, Gelbert L, Talbott R, et al. (1999) PA26, a novel target of the TP53 tumor suppressor and member of the GADD family of DNA damage and growth arrest inducible genes. Oncogene 18: 127-137.

51. Oshiro MM, Futscher BW, Lisberg A, Wozniak RJ, Klimecki WT, et al. (2005) Epigenetic regulation of the cell type-specific gene 14-3-3sigma. Neoplasia 7: 799-808.

52. Fortin A, MacLaurin JG, Arbour N, Cregan SP, Kushwaha N, et al. (2004) The proapoptotic gene SIVA is a direct transcriptional target for the tumor suppressors TP53 and E2F1. J Biol Chem 279: 28706-28714.

53. Hussain SP, Amstad P, He P, Robles A, Lupold S, et al. (2004) TP53-induced up-regulation of MnSOD and GPx but not catalase increases oxidative stress and apoptosis. Cancer Res 64: 2350-2356.

54. Shin TH, Paterson AJ, Kudlow JE (1995) TP53 stimulates transcription from the human transforming growth factor alpha promoter: a potential growth-stimulatory role for TP53. Mol Cell Biol 15: 4694-4701.

55. Wu GS, Burns TF, McDonald ER, 3rd, Jiang W, Meng R, et al. (1997) KILLER/DR5 is a DNA damage-inducible TP53-regulated death receptor gene. Nat Genet 17: 141-143.

56. Takimoto R, El-Deiry WS (2000) Wild-type TP53 transactivates the KILLER/DR5 gene through an intronic sequence-specific DNA-binding site. Oncogene 19: 1735-1743.

57. Shetty S, Graham BA, Brown JG, Hu X, Vegh-Yarema N, et al. (2005) Transcription factor NF-kappaB differentially regulates death receptor 5 expression involving histone deacetylase 1. Mol Cell Biol 25: 5404-5416.

58. Sheikh MS, Huang Y, Fernandez-Salas EA, El-Deiry WS, Friess H, et al. (1999) The antiapoptotic decoy receptor TRID/TRAIL-R3 is a TP53-regulated DNA damage-inducible gene that is overexpressed in primary tumors of the gastrointestinal tract. Oncogene 18: 4153-4159.

59. Liu X, Yue P, Khuri FR, Sun SY (2005) Decoy receptor 2 (DcR2) is a TP53 target gene and regulates chemosensitivity. Cancer Res 65: 9169-9175.

60. Polyak K, Xia Y, Zweier JL, Kinzler KW, Vogelstein B (1997) A model for TP53-induced apoptosis. Nature 389: 300-305.

61. Okamura S, Arakawa H, Tanaka T, Nakanishi H, Ng CC, et al. (2001) TP53DINP1, a TP53-inducible gene, regulates TP53-dependent apoptosis. Mol Cell 8: 85-94.

62. Vossio S, Palescandolo E, Pediconi N, Moretti F, Balsano C, et al. (2002) DN-p73 is activated after DNA damage in a TP53-dependent manner to regulate TP53-induced cell cycle arrest. Oncogene 21: 3796-3803.

63. Sax JK, El-Deiry WS (2003) Identification and characterization of the cytoplasmic protein TRAF4 as a TP53-regulated proapoptotic gene. J Biol Chem 278: 36435-36444.

64. Israeli D, Tessler E, Haupt Y, Elkeles A, Wilder S, et al. (1997) A novel TP53-inducible gene, PAG608, encodes a nuclear zinc finger protein whose overexpression promotes apoptosis. Embo J 16: 4384-4392.

65. Braastad CD, Leguia M, Hendrickson EA (2002) Ku86 autoantigen related protein-1 transcription initiates from a CpG island and is induced by TP53 through a nearby TP53 response element. Nucleic Acids Res 30: 1713-1724.

66. Kurz EU, Cole SP, Deeley RG (2001) Identification of DNA-protein interactions in the 5' flanking and 5' untranslated regions of the human multidrug resistance protein (MRP1) gene: evaluation of a putative antioxidant response element/AP-1 binding site. Biochem Biophys Res Commun 285: 981-990.

67. Wilson LA, Gemin A, Espiritu R, Singh G (2005) ets-1 is transcriptionally up-regulated by H2O2 via an antioxidant response element. Faseb J 19: 2085-2087.

68. Tsuji Y (2005) JunD activates transcription of the human ferritin H gene through an antioxidant response element during oxidative stress. Oncogene 24: 7567-7578.

69. Hintze KJ, Theil EC (2005) DNA and mRNA elements with complementary responses to hemin, antioxidant inducers, and iron control ferritin-L expression. Proc Natl Acad Sci U S A 102: 15048-15052.

70. Mulcahy RT, Wartman MA, Bailey HH, Gipp JJ (1997) Constitutive and beta-naphthoflavone-induced expression of the human gamma-glutamylcysteine synthetase heavy subunit gene is regulated by a distal antioxidant response element/TRE sequence. J Biol Chem 272: 7445-7454.

71. Moinova HR, Mulcahy RT (1998) An electrophile responsive element (EpRE) regulates beta-naphthoflavone induction of the human gamma-glutamylcysteine synthetase regulatory subunit gene. Constitutive expression is mediated by an adjacent AP-1 site. J Biol Chem 273: 14683-14689.

72. Arinze IJ, Kawai Y (2005) Transcriptional activation of the human Galphai2 gene promoter through nuclear factor-kappaB and antioxidant response elements. J Biol Chem 280: 9786-9795.

73. Banning A, Deubel S, Kluth D, Zhou Z, Brigelius-Flohe R (2005) The GI-GPx gene is a target for NRF2. Mol Cell Biol 25: 4914-4923.

74. Montano MM, Deng H, Liu M, Sun X, Singal R (2004) Transcriptional regulation by the estrogen receptor of antioxidative stress enzymes and its functional implications. Oncogene 23: 2442-2453.

75. Onishi Y, Kiyama R (2003) Interaction of NF-E2 in the human beta-globin locus control region before chromatin remodeling. J Biol Chem 278: 8163-8171.

76. Mignotte V, Eleouet JF, Raich N, Romeo PH (1989) Cis- and trans-acting elements involved in the regulation of the erythroid promoter of the human porphobilinogen deaminase gene. Proc Natl Acad Sci U S A 86: 6548-6552.

77. Li Y, Jaiswal AK (1992) Regulation of human NAD(P)H:quinone oxidoreductase gene. Role of AP1 binding site contained within human antioxidant response element. J Biol Chem 267: 15097-15104.

78. Lesniak W, Szczepanska A, Kuznicki J (2005) Calcyclin (S100A6) expression is stimulated by agents evoking oxidative stress via the antioxidant response element. Biochim Biophys Acta 1744: 29-37.

79. Wang Y, Xiao L, Thiagalingam A, Nelkin BD, Casero RA, Jr. (1998) The identification of a cis-element and a trans-acting factor involved in the response to polyamines and polyamine analogues in the regulation of the human spermidine/spermine N1-acetyltransferase gene transcription. J Biol Chem 273: 34623-34630.

80. Boulanger L, Sabatino DE, Wong EY, Cline AP, Garrett LJ, et al. (2002) Erythroid expression of the human alpha-spectrin gene promoter is mediated by GATA-1- and NF-E2-binding proteins. J Biol Chem 277: 41563-41570.

81. Yaekashiwa M, Wang LH (2002) Transcriptional control of the human thromboxane synthase gene in vivo and in vitro. J Biol Chem 277: 22497-22508.

82. Munzel PA, Schmohl S, Buckler F, Jaehrling J, Raschko FT, et al. (2003) Contribution of the Ah receptor to the phenolic antioxidant-mediated expression of human and rat UDP-glucuronosyltransferase UGT1A6 in Caco-2 and rat hepatoma 5L cells. Biochem Pharmacol 66: 841-847.

83. Liu YW, Arakawa T, Yamamoto S, Chang WC (1997) Transcriptional activation of human 12-lipoxygenase gene promoter is mediated through Sp1 consensus sites in A431 cells. Biochem J 324 ( Pt 1): 133-140.

84. Harant H, Eldershaw SA, Lindley IJ (2001) Human macrophage inflammatory protein-3alpha/CCL20/LARC/Exodus/SCYA20 is transcriptionally upregulated by tumor necrosis factor-alpha via a non-standard NF-kappaB site. FEBS Lett 509: 439-445.

85. Wood LD, Richmond A (1995) Constitutive and cytokine-induced expression of the melanoma growth stimulatory activity/GRO alpha gene requires both NF-kappa B and novel constitutive factors. J Biol Chem 270: 30619-30626.

86. Tian B, Nowak DE, Jamaluddin M, Wang S, Brasier AR (2005) Identification of direct genomic targets downstream of the nuclear factor-kappaB transcription factor mediating tumor necrosis factor signaling. J Biol Chem 280: 17435-17448.

87. Fujioka S, Niu J, Schmidt C, Sclabas GM, Peng B, et al. (2004) NF-kappaB and AP-1 connection: mechanism of NF-kappaB-dependent regulation of AP-1 activity. Mol Cell Biol 24: 7806-7819.

88. Figueiredo MS, Brownlee GG (1995) cis-acting elements and transcription factors involved in the promoter activity of the human factor VIII gene. J Biol Chem 270: 11828-11838.

89. Hou J, Baichwal V, Cao Z (1994) Regulatory elements and transcription factors controlling basal and cytokine-induced expression of the gene encoding intercellular adhesion molecule 1. Proc Natl Acad Sci U S A 91: 11641-11645.

90. Xiao W, Hodge DR, Wang L, Yang X, Zhang X, et al. (2004) Co-operative functions between nuclear factors NFkappaB and CCAT/enhancer-binding protein-beta (C/EBP-beta) regulate the IL-6 promoter in autocrine human prostate cancer cells. Prostate 61: 354-370.

91. Harant H, de Martin R, Andrew PJ, Foglar E, Dittrich C, et al. (1996) Synergistic activation of interleukin-8 gene transcription by all-trans-retinoic acid and tumor necrosis factor-alpha involves the transcription factor NF-kappaB. J Biol Chem 271: 26954-26961.

92. Harada H, Takahashi E, Itoh S, Harada K, Hori TA, et al. (1994) Structure and regulation of the human interferon regulatory factor 1 (IRF-1) and IRF-2 genes: implications for a gene network in the interferon system. Mol Cell Biol 14: 1500-1509.

93. Kumar A, Michael P, Brabant D, Parissenti AM, Ramana CV, et al. (2005) Human serum from patients with septic shock activates transcription factors STAT1, IRF1, and NF-kappaB and induces apoptosis in human cardiac myocytes. J Biol Chem 280: 42619-42626.

94. Rein T, Muller M, Zorbas H (1994) In vivo footprinting of the IRF-1 promoter: inducible occupation of a GAS element next to a persistent structural alteration of the DNA. Nucleic Acids Res 22: 3033-3037.

95. Paul NL, Lenardo MJ, Novak KD, Sarr T, Tang WL, et al. (1990) Lymphotoxin activation by human T-cell leukemia virus type I-infected cell lines: role for NF-kappa B. J Virol 64: 5412-5419.

96. Yang H, Sadda MR, Yu V, Zeng Y, Lee TD, et al. (2003) Induction of human methionine adenosyltransferase 2A expression by tumor necrosis factor alpha. Role of NF-kappa B and AP-1. J Biol Chem 278: 50887-50896.

97. Yamamoto K, Arakawa T, Ueda N, Yamamoto S (1995) Transcriptional roles of nuclear factor kappa B and nuclear factor-interleukin-6 in the tumor necrosis factor alpha-dependent induction of cyclooxygenase-2 in MC3T3-E1 cells. J Biol Chem 270: 31315-31320.

98. Zhang Y, Pasparakis M, Kollias G, Simons M (1999) Myocyte-dependent regulation of endothelial cell syndecan-4 expression. Role of TNF-alpha. J Biol Chem 274: 14786-14790.

99. Whelan J, Ghersa P, Hooft van Huijsduijnen R, Gray J, Chandra G, et al. (1991) An NF kappa B-like factor is essential but not sufficient for cytokine induction of endothelial leukocyte adhesion molecule 1 (ELAM-1) gene transcription. Nucleic Acids Res 19: 2645-2653.

100. Hou B, Eren M, Painter CA, Covington JW, Dixon JD, et al. (2004) Tumor necrosis factor alpha activates the human plasminogen activator inhibitor-1 gene through a distal nuclear factor kappaB site. J Biol Chem 279: 18127-18136.

101. Wu H, Lozano G (1994) NF-kappa B activation of TP53. A potential mechanism for suppressing cell growth in response to stress. J Biol Chem 269: 20067-20074.

102. Schwenzer R, Siemienski K, Liptay S, Schubert G, Peters N, et al. (1999) The human tumor necrosis factor (TNF) receptor-associated factor 1 gene (TRAF1) is up-regulated by cytokines of the TNF ligand family and modulates TNF-induced activation of NF-kappaB and c-Jun N-terminal kinase. J Biol Chem 274: 19368-19374.

103. Lai WS, Thompson MJ, Blackshear PJ (1998) Characteristics of the intron involvement in the mitogen-induced expression of Zfp-36. J Biol Chem 273: 506-517.
