# Supplementary material for: Divergent Evolution of Human p53 Binding Sites: Cell Cycle Versus Apoptosis
Source: PLoS Genet. 2007 Jul 27;3(7):e127. doi: 10.1371/journal.pgen.0030127 (PMC1934401; doi:10.1371/journal.pgen.0030127)
Supplement: Table S2 — (152 KB DOC) [file pgen.0030127.st002.doc]

**Table S2: Coordinates of validated TFBSs**

| **Target gene** | **Sequence** | **Coordinates*** | **Strand**** | **Target gene** | **Sequence** | **Coordinates^1^** | **Strand^2^** |
| --- | --- | --- | --- | --- | --- | --- | --- |
| **TP53 TFBSs^3^** | | | | | | | |
| *APAF1* | AGACATGTCT(13)CGACAAGCCC | chr12:97540942-97540974 | +1 | BTG2* | AGTCCGGGCA(1)AGCCCGAGCA | chr1:200006295-200006315 | +1 |
| *BAX** | tcacaagtta(1)agacaagcct | chr19:54149513-54149533 | +1 | CDKN1A | aaacatgccc(11)caacaagctg | chr6:36750474-36750504 | +1 |
| *BAX** | agacaagcctgggcgtgggc | chr19:54149524-54149543 | +1 | CDKN1A* | gaagaagactgggcatgtct | chr6:36753086-36753105 | +1 |
| *BBC3** | CTGCAAGTCCTGACTTGTCC | chr19:52426417-52426436 | -1 | CDKN1A* | gaacatgtcccaacatgttg | chr6:36752204-36752223 | +1 |
| *BID* | gggcatgatggtgcatgcct | chr22:16614037-16614056 | -1 | DUSP1* | gaacttgtca(2)ggctttgttt | chr5:172130047-172130068 | -1 |
| *CASP1* | agacatgcatatgcatgcac | chr11:104411147-104411166 | -1 | GDF15 | catcttgcccagacttgtct | chr19:18357118-18357137 | +1 |
| *CASP6* | aggcaaggag(4)agacaagtct | chr4:110976236-110976259 | -1 | GDF15 | agccatgcccgggcaagaac | chr19:18357996-18358015 | +1 |
| *CTSD** | aagctgggccgggctgaccc | chr11:1741923-1741942 | -1 | MDM2* | ggtcaagttcagacacgttc | chr12:67488970-67488989 | +1 |
| *FOS* | gcgtgcgctc(1)gagcaagtcc | chr14:74815730-74815750 | -1 | MDM2* | gagttaagtc(1)tgacttgtct | chr12:67489007-67489027 | +1 |
| *FOS* | acgcttgccatagtaagaat | chr14:74815751-74815770 | +1 | MLH1 | AGGCATGTAC(2)CGCATGCCCA | chr3:37010251-37010272 | +1 |
| *DCC1* | CAGCATGTTCACACAAGCCA | chr18:48118859-48118878 | +1 | PLK2 | AGACATGGTG(3)AAACTAGCTT | chr5:57794080-57794102 | -1 |
| *FAS** | ggacaagccctgacaagcca | chr10:90741046-90741065 | +1 | PLK2 | AAACATGCCTGGACTTGCCC | chr5:57793858-57793877 | -1 |
| *IGFBP3** | aaacaagcca(1)caacatgctt | chr7:45730921-45730941 | -1 | PLK2 | GGTCATGATT(3)TAACTTGCCT | chr5:57793125-57793147 | -1 |
| *IGFBP3** | GGGCAAGACCTGCCAAGCCT | chr7:45730002-45730021 | -1 | PLK3 | TAACATGCCC(6)AAGCGAGCGC | chr1:44934689-44934714 | +1 |
| *LRDD** | AGGCCTGCCT(8)GGACATGTCT | chr11:794414-794441 | -1 | PTEN | gagcaagccc(14)gggcatgctc | chr10:89613057-89613090 | +1 |
| *P53AIP1** | TCTCTTGCCCGGGCTTGTCG | chr11:128316011-128316030 | -1 | SESN1 | GGACAAGTCTCCACAAGTCT | chr6:109436914-109436933 | -1 |
| *PCBP4** | gaacttaaga(10)ggacaagttg | chr3:51972515-51972544 | -1 | SFN | TAGCATTAGC(2)AGACATGTCC | chr1:26871961-26871982 | +1 |
| *PERP** | AGGCAAGCTCCAGCTTGTTC | chr6:138470600-138470625 | -1 | TP73* | gggcaagctgaggcctgccc | chr1:3630317-3630336 | +1 |
| *PLAGL1* | CAACTAGAC(4)TAGACTAGCTT | chr6:144311857-144311876 | -1 | DKK1 | agccaagctt(6)aaccaagttc | chr10:53741910-53741935 | +1 |
| *PMAIP1** | GAGCGTGTCCGGGCAGGTCG | chr18:55718023-55718042 | +1 | EDN2 | CTGCAAGCCCGGGCATGCCC | chr1:41617174-41617193 | -1 |
| *SOD2* | gtgcttgttc(4)gggcatgtcc | chr6:160086755-160086779 | -1 | FLT1* | GGACACGCTC(5)GGACCTGAGC | chr13:27967923-27967947 | -1 |
| *TNFRSF10B** | GGGCATGTCCGGGCAAGACG | chr8:22982080-22982099 | +1 | NDRG1 | CCACATGCAC(12)GCACATGAAC | chr8:134379022-134379053 | -1 |

| *TNFRSF10C* | gggcatgtccgggcaggacg | chr8:23016747-23016766 | +1 | SERTAD1 | GGGCATGCGCCCTGAAGCCC | chr19:45623874-45623893 | -1 |
| --- | --- | --- | --- | --- | --- | --- | --- |
| *TP53I3** | CAGCTTGCCCACCCATGCTC | chr2:24219686-24219705 | +1 | TGFA | GGGCAGGCCCTGCCTAGTCT | chr2:70692650-70692669 | -1 |
| *TP53INP1** | GAACTTGGGGGAACATGTTT | chr8:96020186-96020205 | -1 | DDB2* | AAGCTGGTTTGAACAAGCCC | chr11:47193096-47193115 | +1 |
| *TRAF4* | gggcaagccagggcctgcct | chr17:24094291-24094310 | +1 | GADD45A* | GAACATGTCTAAGCATGCTG | chr1:67864479-67864498 | +1 |
| *SIVA* | ctacatgcacacgcatgcat | chr14:103984243-103984262 | -1 | PCNA* | ACATATGCCCGGACTTGTTC | chr20:5048821-5048840 | -1 |
| *SIVA* | GTACTTGGCAGGGCATGTCT | chr14:103985698-103985717 | -1 | PMS2 | ATACTTGATT(2)TTTCTTGTAA | chr7:5,818,917-5,818,937 | -1 |
| *SIVA* | gcacaagcct(5)tgtctggagg | chr14:103982439-103982463 | +1 | RRM2B* | tgacatgcccaggcatgtct | chr8:103318244-103318263 | -1 |
| *WIG1** | aaacaagtccagacatgcct | chr3:180270678-180270697 | -1 | XRCC5* | gaactagttt(1)aaacatgttc | chr2:216781166-216781186 | +1 |
| *ADARB1* | GTGCAAGTTTCAACTTGTCC | chr21:45316682-45316701 | -1 | ATF3 | AGTCATGCCG(17)GGTCATGCCT | chr1:209170119-209170155 | 1 |
| *ARHGEF7* | AAACATGTCAGCACTTGCTT | chr13:110602821-110602840 | 1 | C12ORF5 | agacaagtct(2)ggacatgtct | chr12:4301042-4301063 | 1 |
| *DUSP5* | GCACTAGACA(1)GGGCTTGTTG | chr10:112246487-112246507 | -1 | PHLDA3 | ggtcaagttc(6)cagcaggcca | chr1:198169241-198169266 | -1 |
| *PDGFC* | GGTCATGTTCAGACTTGCCC | chr4:158050367-158050386 | -1 | PRKAB1 | gttcttgccgcggcttgcct | chr12:118568545-118568564 | 1 |
| *PPM1J* | GAACATGCCTGAGCAAGCCC | chr1:112958581-112958600 | -1 | SCGB1D2 | GGTCTTGTTTAGACTTGCTC | chr11:61765841-61765860 | 1 |
| *DSC3* | GAAGTTGCTC(6)AGGCAAGCCT | chr18:26876754-26876779 | -1 | SERPINE1 | ACACATGCCTCAGCAAGTCC | chr7:100363660-100363679 | 1 |
| *MASPIN1* | GAACATGTTG(1)AGGCCTTTTG | chr18:59294974-59294994 | 1 | DDIT4 | AAACAAGTCTTTCCTTGATC | chr10:73703380-73703399 | 1 |
| *MASPIN1* | CAAGCTGCCA(2)AGGCTTGAGT | chr18:59295049-59295070 | 1 | FDXR | gggcaggagcgggcttgccc | chr17:70380716-70380735 | -1 |
| *MMP2** | agacaagcctgaacttgtct | chr16:54068958-54068977 | 1 | GPX1 | gggccagaccagacatgcct | chr3:49370958-49370977 | -1 |
| *SEMA3B* | ttgcatgccc(2)agacatgtct | chr3:50280796-50280817 | 1 | SCARA3 | GGGCAAGCCCAGACAAGTTG | chr8:27564569-27564588 | 1 |
| *EOMES* | GGGCCTGTCT(1)CAACTTGCCC | chr3:27739623-27739643 | -1 | RPS27L | GGGCATGTAGTGACTTGCCC | chr15:61236487-61236506 | -1 |
| *UBTD1* | GAGCAAGCCCAGACTTGTCA | chr10:99299918-99299937 | 1 |  |  |  |  |
| **NRF2 TFBSs^4^** | | | | | | | |
| *ABCC1* | TCTGTGTGACTCAGCTTTGGA | chr16:15950431-15950451 | +1 | *HMBS* | CTCCAGTGACTCAGCACAGGT | chr11:118463736-118463756 | +1 |
| *ETS1* | agcggGTGACCAAGCCCTCAA | chr11:127897797-127897817 | -1 | *NQO1* | TCACAGTGACTCAGCAGAATC | chr16:68318405-68318425 | -1 |
| *FTH1* | cctccaTGACAAAGCActttt | chr11:61496126-61496146 | -1 | *S100A6* | GACACGTGACTCGGCAAGGGG | chr1:150321817-150321837 | +1 |
| *FTL* | tcagcatgactcagcagtcgc | chr19:54159005-54159025 | +1 | *SAT* | CCGCTATGACTAAGCGCTAGT | chrX:23559431-23559451 | +1 |
| *GCLC* | TCCCCGTGACTCAGCGCTTTG | chr6:53521000-53521020 | +1 | *SPTA1* | ACTGGGTGACTCAGCAGTTTT | chr1:155469612-155469632 | +1 |
| *GCLM* | TAACGGTTACGAAGCACTTTC | chr1:94087057-94087077 | -1 | *TBXAS1* | AAGGAATGAATCAGCAACTTT | chr7:138982143-138982163 | -1 |
| *GCLM* | AGACAATGACTAAGCAGAAAT | chr1:94087027-94087047 | +1 | *TXNRD1* | TCAGAATGACAAAGCAGAAAT | chr12:103000000-103000000 | -1 |
| *GNAI2* | AGCCTGTGACTGGGCCGGGGC | chr3:50248549-50248569 | -1 | *TXNRD1* | TCATTCTGACTCTGGCAGTTA | chr12:103183181-103183201 | +1 |
| *GPX2* | CCAGGATGACTTAGCAAAAAC | chr14:64479324-64479344 | +1 | *UGT1A6* | TCTGTCTGACTTGGCAAAAAT | chr2:234383053-234383073 | -1 |
| *GSTP1* | gcgccgtgactcagcactggg | chr11:67107787-67107807 | +1 | *UGT1A6* | GAAAGCTGACACGGCCATAGT | chr2:234383432-234383452 | +1 |
| *HBB* | TCATCATGACTCAGCATTGCT | chr11:5258563-5258583 | +1 |  |  |  |  |
| **NFKB TFBSs^5^** | | | | | | | |
| *SELE* | GGGGATTTCC | chr1:166434963-166434972 | -1 | *TRAF1* | GGGGAACTCT | chr9:120768238-120768247 | -1 |
| *PTGS2* | GGGGATTCCC | chr1:183381652-183381661 | -1 | *TNIP1* | GGAAAGTCCC | chr5:150440821-150440830 | -1 |
| *MAT2A* | GGGAGGTGCC | chr2:85677603-85677612 | 1 | *NFKBIA* | GGAAATTCCC | chr14:34943778-34943787 | -1 |
| *CCL20* | GGGGAAAACC | chr2:228503982-228503991 | 1 | *ALOX12* | GGGACATCCC | chr17:6839623-6839632 | 1 |
| *IL8* | TGGAATTTCC | chr4:74971229-74971238 | 1 | *TP53* | GGGGTTTTCC | chr17:7531575-7531584 | -1 |
| *CXCL1* | GGGAATTTCC | chr4:75100066-75100075 | 1 | *ICAM1* | GGAAATTCCG | chr19:10242610-10242619 | 1 |
| *IRF1* | GGGAATCCCG | chr5:131854404-131854413 | -1 | *ZFP36* | GGGGATCCGA | chr19:44589788-44589797 | 1 |
| *LTA* | GGGGCTTCCC | chr6:31647942-31647951 | 1 | *SDC4* | GGGGAATTCC | chr20:43410566-43410575 | -1 |
| *IL6* | TGGGATTTTC | chr7:22539985-22539994 | 1 | *ELK1* | GGGAAGGGCC | chrX:47266341-47266350 | -1 |
| *SERPINE1* | TGGAATTTCT | chr7:100349129-100349138 | 1 | *F8* | GGGACTTTTC | chrX:153814635-153814644 | -1 |
| *TNFRSF10B* | GGGAATTCCC | chr8:22981940-22981949 | 1 |  |  |  |  |

*Member of the most-well known TP53 RE list utlized in figure S1

^1^Relative to UCSC genome browser, Human genome build 35.1 (May 2004 release), 1-based coordinates

^2^+1 = forward strand, -1 = reverse strand

^3^TP53 consensus sequence = RRRCWWGYYY(N_0-13_)RRRCWWGYYY. The number of spacer bases are shown in parentheses.

^4^NRF2 consensus sequence = TMAnnRTGAYnnnGCRWWW

^5^NFKB consensus sequence = GGGRnnYYCC
