# Supplementary material for: Divergent Evolution of Human p53 Binding Sites: Cell Cycle Versus Apoptosis
Source: PLoS Genet. 2007 Jul 27;3(7):e127. doi: 10.1371/journal.pgen.0030127 (PMC1934401; doi:10.1371/journal.pgen.0030127)
Supplement: Table S3 — (39 KB DOC) [file pgen.0030127.st003.doc]

**Table S3:** Correlation between RE identity distributions and distributions obtained from random sampling of promoter fragments. Paired distributions of the identity bins from Figure 2 were converted to percentages and a correlation between the RE distribution and promoter sample distribution was determined. A high correlation indicates that the shapes of the distributions are similar. p53 REs show high correlation with sample of promoter fragments. NRF2 and NFKB show lower correlation with promoter fragments.

| **Transcription factor binding site distribution** | **Comparison distribution** | **Correlation Coefficient** | | | |
| --- | --- | --- | --- | --- | --- |
|  |  | **Mouse** | **Rabbit** | **Rat** | **Dog** |
| **p53** | promoter fragments | 0.68 | 0.92 | 0.79 | 0.87 |
|  |  |  |  |  |  |
| **NRF2** | promoter fragments | 0.09 | 0.79 | 0.10 | 0.65 |
|  |  |  |  |  |  |
| **NFKB** | promoter fragments | 0.09 | 0.46 | 0.24 | 0.36 |
